# Substitution patterns and price response for plant-based meat alternatives

**DOI:** 10.1073/pnas.2319016121

**Published:** 2024-12-02

**Authors:** Steffen Jahn, Daniel Guhl, Ainslee Erhard

**Affiliations:** ^a^Department of Business Administration, School of Economics and Business, Martin Luther University Halle-Wittenberg, Halle 06099, Germany; ^b^Department of Business Administration, School of Business and Economics, Humboldt University Berlin, Berlin 10099, Germany; ^c^Department of Business Administration, Faculty of Business and Economics, University of Göttingen, Göttingen 37073, Germany

**Keywords:** plant-based meat, food decision-making, sustainability, price elasticity, meat substitute

## Abstract

Efforts to promote sustainable resource use through reduced meat consumption face challenges as global meat consumption persists. The resistance may be attributed to the lower sales price of meat compared to most plant-based meat alternatives (PBMAs). Addressing this, our research delves into the pivotal question of which PBMAs resonate most with consumers and how pricing affects demand. In a hypothetical restaurant context, we conducted 2 representative studies among 2,126 individuals in the United States to scrutinize preferences for meat, analog, semi-analog, and non-analog burgers. First, in a survey, we assessed rankings of the four burgers, alongside evaluating participants’ genuine consideration of these choices to discern a diverse preference distribution. Subsequently, in an experiment, we examined the influence of prices on participants’ consideration and choice of PBMAs, thereby capturing both phases of the decision-making process. Our survey shows that meat has considerably higher utility and consumer preference than all PBMAs on average, but we also find substantial heterogeneity (i.e., some consumers prefer PBMAs over meat). In the experiment, we establish that there is a negative association between the consideration of meat and PBMA burgers, though consideration of any one PBMA is positively associated with considering other PBMAs. A noteworthy increase in consideration and choice is observed when prices of PBMAs are reduced, while changing the price of the meat burger only has minimal effect on demand. Such findings underscore the importance of affordability beyond price parity in catalyzing the shift toward plant-based diets.

## Significance

Diminishing meat intake in wealthier nations is crucial for environmental preservation and public health. Plant-based meat alternatives (PBMAs) can help accomplish this goal, yet limited research explores complex substitution patterns and the influence of price adjustments. Further, we are aware of no studies examining the impact of price variation at the consideration and choice stage. Through robust survey and experimental research, we demonstrate that meat has considerably higher utility than PBMAs yet demand for PBMAs is sizeable, especially among certain consumer types. Individuals who begin to consider one PBMA type are more likely to weigh others. Lowering PBMA prices emerges as a strategic lever, offering a potential avenue to achieve the outcome of heightened consideration and choice of sustainable food.

Scientists agree that decreasing consumption of meat, particularly in wealthier nations, is an effective means toward sustainable use of global resources (1, 2). Yet global meat consumption shows few signs of decline (3). To some extent, this can be attributed to insufficient supply and/or promotion of attractive plant-based meat alternatives (hereafter PBMAs). Accordingly, a central question is which kind of PBMAs will resonate the most with consumers and how affordability can facilitate demand.

In a systematic review on consumer acceptance of alternative proteins, Onwezen and colleagues (4) concluded that consumers are more willing to accept plant-based novel proteins than animal-based novel proteins, such as insects and cultured meat. Circus and Robison (5) found that consumers with high (vs. low) meat attachment are more willing to eat meat alternatives but remain skeptical overall. A surprising limitation of previous research is the undifferentiated view of PBMAs. The majority of studies lump together meat alternatives or compare one PBMA with its meat counterpart (4, 6). Importantly, plant-based protein has been around for thousands of years (7). Early vegetarian proteins, such as tofu and tempeh, were mainly consumed by vegetarians (8). These foods bore little resemblance to their meat counterparts, whereas recent advancements have focused on creating plant-based meat analogs that closely mimic the taste and texture of meat proteins, using innovative technologies and ingredients like pea protein and heme (9, 10). Well-known examples of analogs are Beyond Meat and the Impossible Burger; such products are now commonplace in grocery stores as well as fast-food chains such as Burger King and Shake Shack. Recently, British consumer goods giant Unilever partnered with German Düzgün Group to develop a plant-based Döner kebab skewer for sale in kebab shops (11).

Despite any technological advancements, it is debated whether meat-mimicking PBMAs are a fad or the future (12). During the coronavirus pandemic in 2020, for example, Beyond Meat reported a series of better-than-expected quarterly results, but revenue has plummeted ever since. The up and down suggests general interest in meat analogs as novelty-seeking behavior (13) that could not be sustained, perhaps because not all consumers liked the product at the time. Favorable characteristics of meat analogs, such as similar sensory experience and familiar preparation (6), notwithstanding, a study among 2,497 Swedish adult consumers found that lightly processed legumes were seen as more attractive than legume-based meat analogs (14). Despite major interest in the topic of PBMAs, with more than 1,800 published articles in the Web of Science Core Collection as of July 2024 (15), there is limited empirical evidence on how consumers perceive the various existing PBMAs. In response, in this research, we consider three types of PBMAs: i) analog, ii) semi-analog, and iii) non-analog. Analog PBMAs try to mimic meat in every possible way, whereas semi-analog PBMAs can be seen as first-generation alternatives that, like a veggie burger, are analog in general appearance but not taste or texture. Non-analog PBMAs are based on traditional nonmeat dishes and have not been altered to look or feel like meat but can be used in the same consumption contexts.

Beyond examination of different PBMA types, research is only beginning to understand the role prices play in PBMA preference and choice (16, 17). In the United States., for instance, prices for beef alternatives exceed those for beef by 20% (18). In a study among 1,039 German adult consumers, both omnivores and flexitarians mentioned that they perceive meat to perform better than meat alternatives in terms of price (19). Given the high price tag of some of these products, there has been an ongoing discussion on the need to make PBMAs more affordable. Fast-food giant McDonald’s, for instance, withdrew its meat-mimicking McPlant burger from the US market after poor sales across 600 test restaurants (20). Customer feedback indicates that, despite its appealing taste, the McPlant was considered too expensive: “It tastes like real meat, but it’s too expensive to buy again” (21). Price may partly explain why studies find PBMA market shares to be limited in the 20-25% range (16, 17, 22). In response, grocery chain Lidl in Germany launched an ambitious initiative to achieve price parity between PBMAs and their meat counterparts (23). However, it raises a pivotal question: Can achieving price parity alone instigate a significant shift in protein consumption, or is it imperative for PBMAs to surpass meat in affordability? Because plant-based protein has lower per-gram costs than meat (7), greater affordability appears an attainable scenario as soon as processing costs have decreased.

To tackle these research gaps, we conducted 2 large-scale studies—1 survey (N_STUDY1_ = 1,003) and 1 experiment (N_STUDY2_ = 1,123)—to examine preferences for certain PBMAs. In the survey, we considered rankings of a meat burger and three PBMA burgers as well as genuine consideration of these options to determine the heterogenous preference distribution. In the experiment, we tested the impact of price on burger consideration and choice.

## Study 1: Preference Heterogeneity

### Aims and Design.

The goal of this study was to gain a better understanding of preferences for various PBMAs. We showed 1,003 American adults images and patty ingredient lists of four burger alternatives: meat (beef burger), analog (which mimics meat; plant-based burger), semi-analog (analog in general appearance but not taste or texture; veggie burger), and non-analog (falafel burger) (*Methods* and *SI Appendix*, Fig. S1). We asked respondents to rank all four burgers and to indicate, for each burger option, if they would genuinely consider purchasing it outside the study context.

## Results

### Preferences: Model-free Evidence.

The ranking task reveals that, unsurprisingly, the meat burger is by far the most popular option (75.0% of first-place votes; *SI Appendix*, Fig. S2). The burger with the second-highest share of first-place votes (11.5%), as well as the largest share of second-place votes (33.7%), is the non-analog falafel burger. The semi-analog and analog—both options that mimic meat, albeit to different degrees—are rated very similarly (7.3% and 6.3% of first-place votes, respectively). The largest share of “last-place votes” is received by the meat analog. Consideration is highest for the meat burger (91%) but is also sizeable for PBMAs, with shares ranging between 48.5% (semi-analog burger) and 43.3% (analog burger). Burger options that rank first are almost always considered (99%), whereas lower ranks have lower consideration (69.4%, 42.3%, and 17.1% for the ranks 2 to 4, respectively). This makes intuitive sense and highlights the value of the data for our analysis, as it provides valuable information about burger preferences beyond first choices.

### Preferences: Model-based Evidence.

**Fig. 1.**
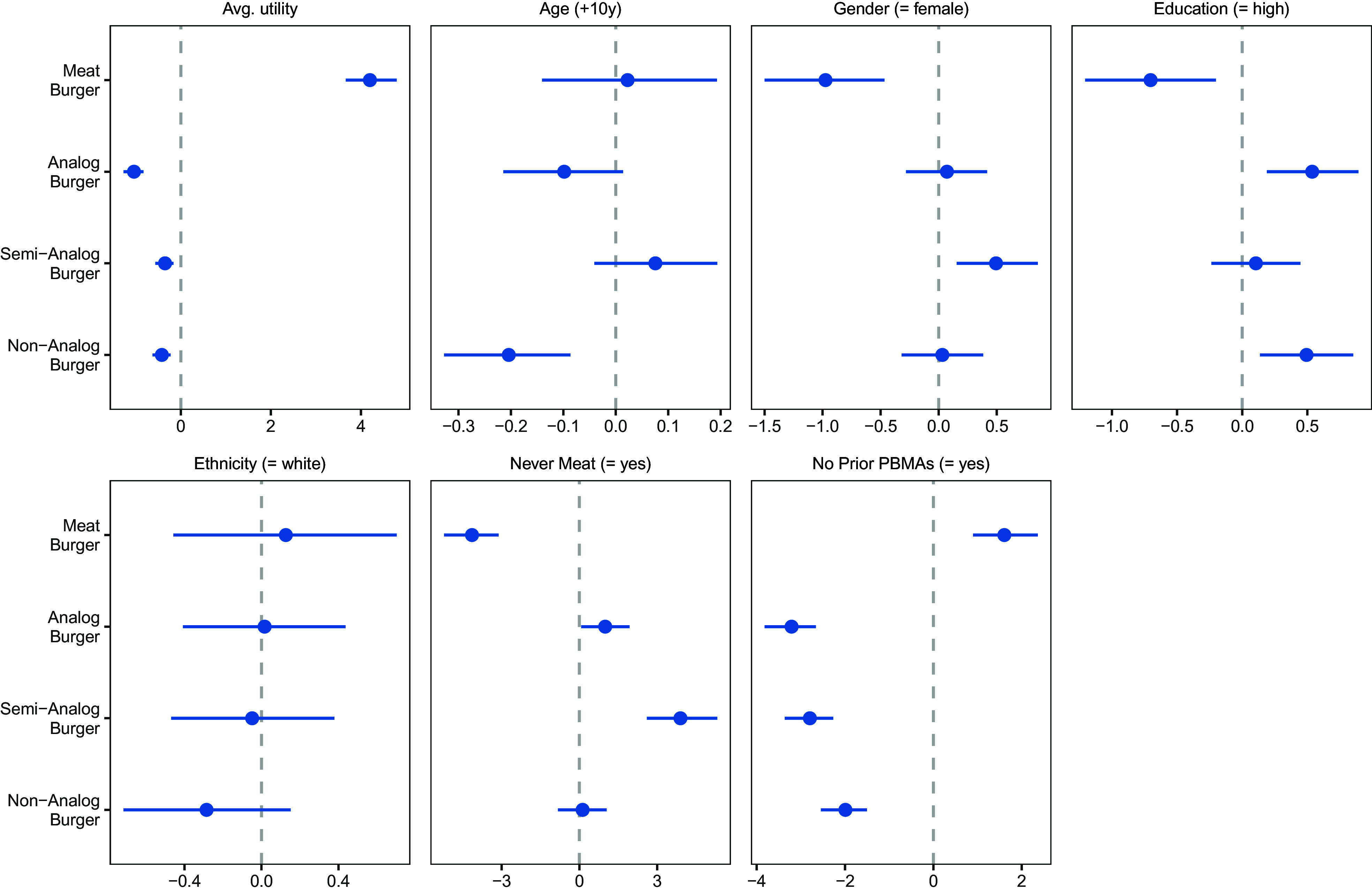
Exemplary Individual Differences in Burger Preference (Study 1). Note: The first box shows the average utility of each burger option that accounts for heterogeneity in preferences of the US population. The remaining boxes show observed heterogeneity as planned contrasts (posterior mean plus 95% credible intervals). Education = high: college degree or higher; Never Meat = yes: self-report to never eat meat; No Prior PBMAs = yes: self-report to never eat plant-based meat alternatives (PBMAs). For example, the meat burger has lower utility for females than males, whereas the semi-analog burger has higher utility for females. Numerical results are shown in *SI Appendix*, Table S2, Panel *A*.

We estimate a hierarchical exploded logit model (24, 25) to incorporate multiple-ranked choices for each person, consideration [in addition to the ranking for anchoring purposes (26)], as well as unobserved and observed heterogeneity (*Methods* and *SI Appendix*, SI Text). Overall, we find that the meat burger has higher utility than all PBMAs (*SI Appendix*, Table S2, Panel *A*). Results further point to preference differences across consumer types (Fig. 1). For example, female respondents have lower utility for the meat burger but higher utility for the semi-analog burger (compared to males). Similarly, high education is associated with lower meat burger utility but higher analog and non-analog burger utility (compared to low education). The non-analog burger has shrinking utility with increasing age (by 10-y cohort).

After controlling for observed heterogeneity, we still find significant *σ* values for all four burgers (*SI Appendix*, Table S2, Panel *B*), which points to substantial unobserved heterogeneity in burger preferences. The magnitudes of the *σ* values of about 1.8 to 2.0 help explain why not all respondents rank the burgers in the same order. Further, for the unobserved heterogeneity, we also estimate correlations between the options’ utilities (*SI Appendix*, Table S2, Panel *C*) and find significantly negative relationships between the meat and non-meat burgers (meat_analog: *ω* = −0.46; meat_semi-analog: *ω* = −0.50; meat_non-analog: *ω* = −0.41). The correlations between the PBMA options are all positive and significant (analog_semi-analog: *ω* = 0.75; analog_non-analog: *ω* = 0.46; semi-analog_non-analog: *ω* = 0.38), pointing to a complementary relationship among the three PBMA types.

### Counterfactual Simulation.

Based on the hierarchical exploded logit model, it is possible to run counterfactual (“what if”) simulations (27). We simulate the market shares for several scenarios with varying availability of burger options while accounting for the estimated preference heterogeneity and consideration effects (*SI Appendix*, SI Text). In a scenario with all four options available (scenario 8 in *SI Appendix*, Table S3), 75.3% of consumers would choose the meat burger, followed by 11.1% choosing the semi-analog, 8.4% the non-analog, and 4.8% the analog burger. We note that these shares align well with the model-free results for the 1st rank shares reported (*SI Appendix*, Fig. S2), lending credibility to the simulation results. The finding suggests that most consumers prefer the original (i.e., meat burger) over the replica (i.e., analog burger) but also that “traditional” PBMAs, such as the semi-analog veggie burger and the non-analog falafel burger, are no less popular than meat analogs. Results further indicate that few respondents choose the none option, meaning that collective demand for the 3 PBMAs is close to 25%. We note that this estimate is remarkably similar to that found in previous studies (16, 17, 28). In contrast to a scenario featuring meat alongside a single PBMA, the introduction of a second and third PBMA indicates, on average, a 42.1% and 17.5% overall increase in market shares for PBMAs. This implies a notable rise in PBMA share (and a decrease in meat share by 5.7% and 4%, respectively) as the choice set expands to include more PBMAs.

A simulation in which the meat option is not available (scenario 15 in *SI Appendix*, Table S3) suggests that market shares would notably increase for each PBMA: analog 15.1% (+215%), semi-analog 27.3% (+146%), and non-analog 26.5% (+215%). Apparently, all PBMAs benefit from the unavailability of meat in nearly the same way, including the semi-analog and non-analog burgers. Although 31.0% would leave the market and choose none of the plant-based proteins, collective PBMA demand would increase to 68.9%. This indicates that, with some incentive, consumers might become more open to PBMAs, almost tripling market shares relative to the baseline scenario.

## Study 2: Price Response

### Aims and Design.

In this discrete choice experiment, we used a 2 (relative price: 25% higher vs. lower than the baseline price of $10) × 4 (burger option) plus control (all prices are at the baseline) between-subjects design to test the impact of price on PBMA consideration as well as choice, aiming to capture both stages of the decision-making process. We aimed for 125 participants per cell due to the anticipated high share of meat burger choice (final N_STUDY2_ = 1,123). We showed participants the same burger options (i.e., meat, analog, semi-analog, and non-analog) as in study 1 and asked them to indicate which they would consider ordering for lunch (*SI Appendix*, Fig. S3). Based on their answer, participants were asked to indicate which of the considered burgers they preferred the most. Asking for consideration and choice separately allows us to better analyze complex substitution patterns (29).

## Results

### Substitution and Price Response: Model-free Evidence.

A descriptive analysis of burger consideration frequencies reveals that most consumers consider the meat burger (85.2%), followed by the non-analog (36.1%), analog (35.8%), and semi-analog burgers (34.8%). In terms of absolute and relative magnitude, these values are similar to those in study 1. A comparison of the empirical distribution of consideration sets and their implied distribution given the aforementioned burger consideration frequencies assuming independence reveals significantly different results (*SI Appendix*, Fig. S4). The discrepancy indicates that accounting for dependencies (negative or positive) between burger consideration is crucial for understanding consumer preferences. The most common consideration set contains only the meat burger (38.4%). Further, consumers tend to consider either one specific PBMA option or all of them, while consideration sets with two plant-based burgers are rare. Similar to what we find in study 1, approximately three-quarters (72.7%) would choose the meat burger, followed by the non-analog (8.3%), analog (8.3%), and semi-analog (6.9%) burgers. In terms of price response, we find that the average consideration for a burger increases by about 9% points at the low price point of $7.50 compared to the high price point of $12.50. Similarly, choice increases by about 5% points when comparing burgers offered at $7.50 versus $12.50. These differences are economically meaningful.

### Substitution: Model-based Evidence.

To combine the consideration and choice stages, we use a two-stage model (30) that first employs a multivariate logit model (31) to analyze burger consideration, allowing for associations between alternatives. In the second stage, we use a multinomial logit model (25) for burger choice, using only the observed consideration sets. In both stages, we account for price effects and observed heterogeneity. Both models fit the data very well, with predicted consideration (choice) shares of 85.1% (72.6%), 36.7% (7.9%), 35.1% (6.2%), and 36.4% (8.1%), for the meat, analog, semi-analog, and non-analog burgers, respectively (the predicted consideration shares also align with the data; *SI Appendix,* Fig. S4). Parameter estimates (*SI Appendix*, Table S6) indicate that female consumers consider the semi-analog burger more than male consumers [θ = 0.69, 95% CI: (0.38, 0.99)] but consider the analog [θ = −0.34, 95% CI: (−0.62, −0.08)] and non-analog burgers [θ = −0.60, 95% CI: (−0.90, −0.31)] less. Highly educated consumers show no differences in PBMA burger consideration but consider the meat burger less than consumers without a college degree [θ = −0.72, 95% CI: (−1.16, −0.28)]. We further observe an uphill battle for PBMA consideration among consumers who self-report to never buy PBMAs. These consumers have higher meat consideration [θ = 1.54, 95% CI: (0.75, 2.44)] and lower plant-based burger consideration, irrespective of PBMA type (θs between −1.41 and −0.63, *P*s < 0.05). Similar to study 1, we find that meat burgers have the highest utility at the choice stage but differences to the PBMAs are less pronounced as we model burger choice conditional on consideration. Parameter estimates (*SI Appendix*, Table S6) show that female consumers have a lower utility for the meat [θ = −0.86, 95% CI: (−1.59, −0.15)] and analog burgers [θ = −0.85, 95% CI: (−1.71, −0.03)]. As with the consideration stage, consumers with no prior PMBA experience have a lower utility for PBMA burgers (θs between −1.64 and −1.22, statistically significant for analog and non-analog burgers).

Echoing substitution patterns from study 1 as well as the model-free results, association patterns between the four burger options indicate that consideration sets that include the meat option may not contain any PBMAs (Table 1, *Lower-left part*). For example, individuals are less likely to consider both the meat and non-analog burgers [ψ = −0.82, 95% CI: (−1.28, −0.37)]. Conversely, consideration of any PBMA is associated with increased consideration of the remaining PBMAs. Positive and significant association parameters are found for the semi-analog and analog [ψ = 0.86, 95% CI: (0.56, 1.16)] as well as non-analog burger [ψ = 1.45, 95% CI: (1.15, 1.77)], respectively. The association between the analog and non-analog burger is nonsignificant [ψ = 0.22, 95% CI: (−0.08, 0.51)]. Apparently, individuals seem to consider these specific PBMAs independently of each other.

**Table 1.** Association Parameters and Correlations (Study 2)

Burger Alternative	Meat Burger	Analog Burger	Semi-Analog Burger	Non-Analog Burger
Meat Burger	1	**−0.32**	**−0.55**	**−0.47**
Analog Burger	**−0.43** [−0.87, −0.01]	1	**0.44**	**0.31**
Semi-Analog Burger	**−0.52** [−1.00, −0.05]	**0.86** [0.56, 1.16]	1	**0.59**
Non-Analog Burger	**−0.82** [−1.28, −0.37]	0.22 [−0.08, 0.51]	**1.45** [1.15, 1.77]	1

Note: Tetrachoric correlations (model-free) are shown in the upper-right part. Association parameters (multivariate logit model) are shown in the lower-left part with 95% credible intervals (CI) in parentheses. The corresponding utility parameters of the multivariate logit model are reported in *SI Appendix*, Table S6. Significant estimates (at 5%) are bolded.

### Price Response: Model-based Evidence.

Price has a positive but nonsignificant effect on meat consideration [θ = 0.17, 95% CI: (−0.08, 0.42)] but a negative and significant effect on PBMA consideration [θ = −0.11, 95% CI: (−0.18, −0.04)] and choice for all options [θ = −0.19, 95% CI: (−0.34, −0.04)] (*SI Appendix*, Table S6). Being older [θ = 0.08, 95% CI: (0.01, 0.15)], identifying as female [θ = 0.29, 95% CI: (0.05, 0.52)], and having a college degree [θ = 0.35, 95% CI: (0.13, 0.58)] significantly decrease price sensitivity regarding burger choices. To interpret the price effect, we examine price elasticities at the consideration stage as well as total elasticities (i.e., consideration and choice). Total elasticities (Table 2, Panel *B*) show how choices among the four burger options plus the option to choose none change depending on price. Own elasticities are negative for all burgers and stronger in absolute terms for PBMAs. Specifically, the elasticity for the analog burger is significant and greater than |−1| (ε = −1.4), indicating price-elastic demand. Reducing the analog burger’s price by 10%, for instance, would result in a 14% sales increase. For the semi-analog and non-analog burgers, demand is price-inelastic (−1 < ε < 0). The price elasticity for the meat burger is only −0.05 [95% CI: (−0.26, 0.16)] and nonsignificant [Pr(ε < 0) = 0.669]. Hence, we do not observe a meaningful effect of price on meat burger choices. By contrast, for any 1-percent decrease (increase) of the sales price of a given PBMA, the choice of that alternative increases (decreases) by approximately 0.5 to 1.4 percent. We find that choice elasticities are comparable to those in previous studies, especially considering our out-of-home food demand context (16, 32–34).

**Table 2.** Price Elasticity Decomposition: Consideration and Total Elasticities (Study 2)

Panel A: Consideration Elasticity				
	Meat Burger	Analog Burger	Semi-Analog Burger	Non-Analog Burger
Meat Burger	0.15 [−0.01, 0.31]	**0.01** [0.00, 0.03]	**0.02** [0.00, 0.04]	**0.02** [0.01, 0.04]
Analog Burger	−0.05 [−0.14, 0.01]	**−0.64** [−1.01, −0.25]	**−0.12** [−0.20, −0.04]	**−0.07** [−0.14, −0.02]
Semi-Analog Burger	−0.07 [−0.18, 0.01]	**−0.12** [−0.21, −0.04]	**−0.56** [−0.91, −0.20]	**−0.19** [−0.32, −0.07]
Non-Analog Burger	−0.09 [−0.20, 0.01]	**−0.07** [−0.14, −0.02]	**−0.18** [−0.31, −0.07]	**−0.64** [−1.02, −0.26]
Outside Good	−1.46 [−3.05, 0.11]	**0.48** [0.15, 0.84]	**0.61** [0.20, 1.11]	**0.55** [0.19, 0.95]
Panel B: Total Elasticity				
	Meat Burger	Analog Burger	Semi-Analog Burger	Non-Analog Burger
Meat Burger	−0.05 [−0.26, 0.16]	**0.13** [0.06, 0.20]	**0.08** [0.03, 0.13]	**0.05** [0.02, 0.09]
Analog Burger	0.39 [−0.27, 1.10]	**−1.39** [−2.31, −0.48]	−0.13 [−0.32, 0.05]	0.00 [−0.09, 0.11]
Semi-Analog Burger	0.00 [−0.57, 0.58]	−0.01 [−0.24, 0.23]	−0.46 [−1.42, −0.49]	0.03 [−0.16, 0.26]
Non-Analog Burger	0.01 [−0.56, 0.59]	0.01 [−0.20, 0.21]	**−0.32** [−0.65, −0.03]	**−0.66** [−1.09, −0.25]
Outside Good	0.09 [−0.89, 1.13]	**0.21** [0.05, 0.38]	0.11 [−0.11, 0.34]	**0.20** [0.06, 0.38]

Note: Elasticity values are shown with 95% credible intervals (CI) in parentheses. Significant elasticities (at 5%) are bolded. Elasticities represent the percentage change in consideration and choice for a burger alternative in a row in response to a 1-percent price increase of an alternative in a column.

Cross-price elasticities indicate that after a PBMA price decrease, the increased PBMA choice comes from individuals who would not have chosen any burger option (i.e., from the outside good) but also from those who choose the meat option when prices are the same across all products (see also ref. 34). The effect, both in terms of pattern and effect size, is similar for each of the three PBMAs, but the analog burger has the highest cross-price elasticity with regard to the meat burger [ε = 0.13, 95% CI: (0.06, 0.20)]. All cross-price elasticities of the meat burger are nonsignificant, but there is an 87.1% probability that increasing the price of the meat burger would increase the choice of the analog burger [ε = 0.39, 95% CI: (−0.27, 1.10)].

In addition to the pattern described above, significant cross-price elasticities among the PBMAs exist at the consideration stage, reflecting the patterns of the association parameters (Table 2, Panel *A*). For example, reducing the price of the semi-analog burger not only increases the likelihood of considering that semi-analog burger (ε = −0.56); it also increases the likelihood of considering the analog (ε = −0.12) as well as non-analog burgers (ε = −0.18). At the same time, consideration of the meat burger becomes slightly less likely [ε = 0.02, 95% CI: (0.00, 0.04)]. Decomposition of the total effect reveals that about half to almost all of the total price effect on PBMA choice comes from the price effect on consideration, hence modeling price effects at both stages is crucial. Both effects, increased consideration of PBMAs and decreased consideration of the meat option, are prerequisites for a sustained behavior change. To understand the total effects of specific price scenarios and differences across consumer types, we look at the counterfactual simulations in the next step.

### Counterfactual Simulations.

**Fig. 2.**
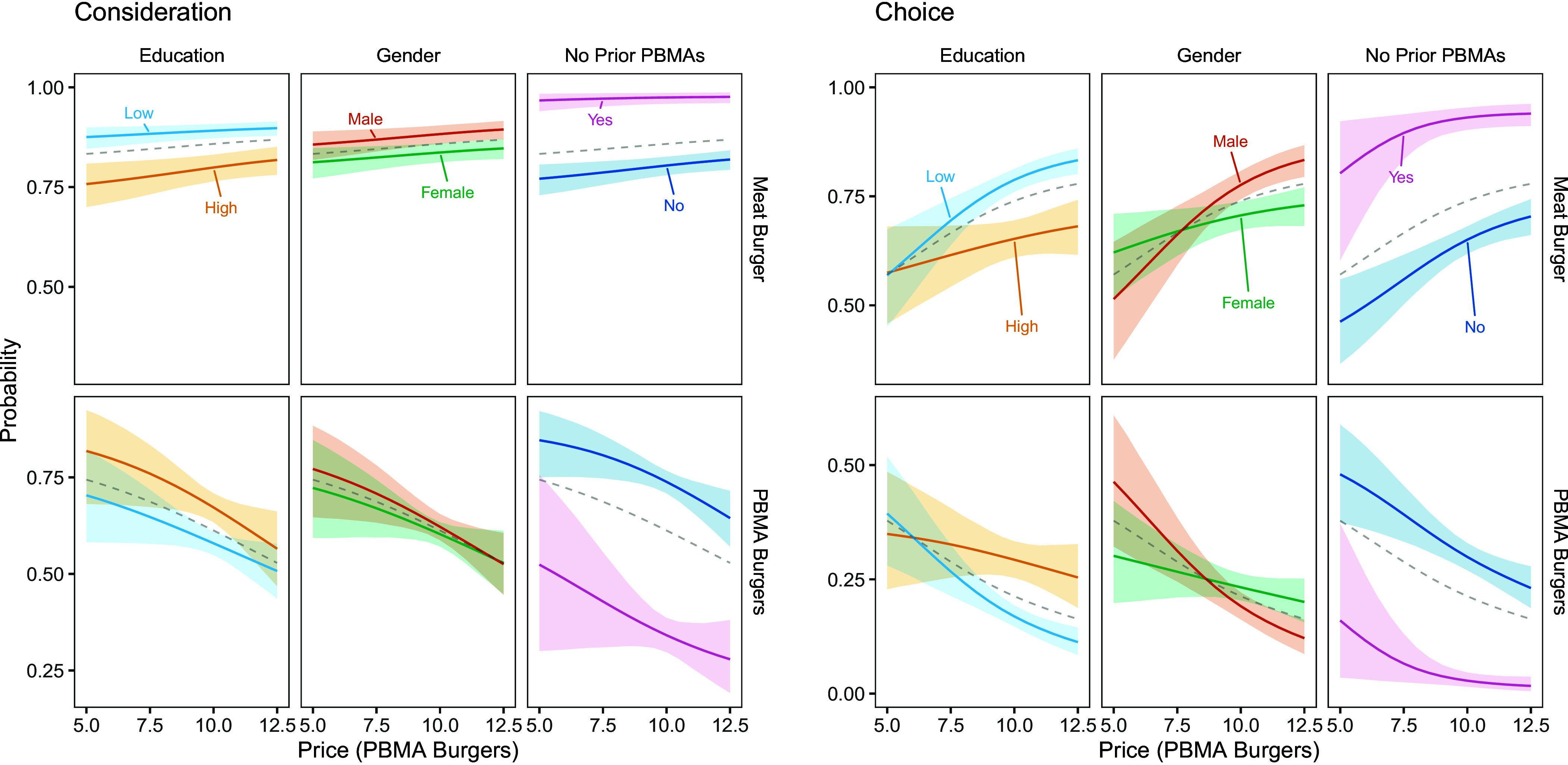
Price Effect Simulations for Meat and PBMA Burgers (Study 2). Note: Consideration and choice probabilities are shown for the meat burger and PBMAs (aggregated) given a PBMA price range between $12.50 and $5, along with 95% credible intervals. Across PBMA price scenarios, the price of the meat burger was kept constant at $10. A PBMA price of $10 thus corresponds to price parity. The gray dashed lines display the population mean (representative of the United States). Education = high: college degree or higher; No Prior PBMAs = yes: self-report to never eat PBMAs. Disaggregated PBMA results are shown in *SI Appendix*, Figs. S5–S7.

To understand price response across different price scenarios and consumer types, we show meat and PBMA consideration and choice in Fig. 2 (effects for individual PBMA types are shown in *SI Appendix*, Figs. S5–S7). We simulate outcomes for different PBMA prices (between $12.50 and $5) and a fixed price for the meat burger ($10). The counterfactuals underscore the price response such that both meat and nonmeat consideration and choice are responsive to price changes, especially large ones. When we compare a scenario of price parity with one in which PBMAs cost half the price of meat, meat burger consideration probability drops by 2.5% points (from 85.8% to 83.3%) and meat choice probability by 17% points (from 73.9% to 57%), while PBMA consideration increases by 13% points (from 61.2% to 74.4%) and choice probability by 16.5% points (from 21.3% to 37.8%). The positive price effect on PBMA consideration is not only visible as an increased likelihood of considering at least 1 PBMA option; we also observe that consideration set size increases (in the baseline condition, only 27.6% of consideration sets include more than 2 burgers; that number increases to 46.6 when the PBMA price decreases).

Further, individual differences point to consumer types that vary in price response (*SI Appendix*, Table S6). For example, highly educated consumers are generally less likely to consider meat and more likely to consider PBMAs (compared to consumers without a college degree, Fig. 2). As PBMA prices become more competitive, however, the gap widens with regard to PBMA consideration but narrows for PBMA choice. Although US consumers without a college degree consider PBMAs less often, lower PBMA prices significantly increase their likelihood of purchasing meat alternatives, narrowing the gap with highly educated consumers.

In terms of gender, males are more likely to consider meat but also PBMAs (a look at the individual PBMA types reveals that males are more likely to consider the analog and non-analog burger but less likely to consider the semi-analog burger; *SI Appendix*, Fig. S5). Despite this general difference in burger consideration, males and females respond similarly to price changes. When it comes to burger choice, however, price affects gender very differently. While males become more likely to choose PBMAs as their price becomes more competitive, a favorable price has a minimal effect on PBMA choice among females (irrespective of PBMA type).

Counterfactuals also illuminate the type of “no prior PBMA eaters.” Individuals who do not normally eat PBMAs seem less normative about it (unlike individuals who state they never eat meat; *SI Appendix*, Fig. S5). This indicates that unfamiliarity with PBMAs does not carry forward; instead, there is an openness to at least consider PBMAs, especially when they become more affordable (Fig. 2). Stimulating PBMA sales in this type remains an uphill battle though. By contrast, individuals with at least some experience of purchasing PBMAs are more willing to refrain from purchasing the meat burger and try PBMAs instead, if the price becomes attractive. When we simulate education and gender effects only within the group of individuals with at least some PBMA experience, we find that males without a college degree would be almost indifferent between a meat and analog burger (each choice probability ~30%) when the latter costs half the price (*SI Appendix*, Fig. S7).

## Discussion

Consumers overwhelmingly prefer meat over PBMAs, yet the landscape becomes less clear when determining which alternatives to meat they might also consider and the conditions under which shifts in preference occur. This study sought to untangle these complexities, providing a deeper understanding of consumer preferences in this domain. Our findings underscore the necessity of differentiating between various categories of PBMAs, given the increasing diversity of options on the market, rather than treating them as a homogeneous group, a distinction often overlooked in existing studies (6). This nuanced approach becomes crucial when examining preferences and substitution patterns.

First, PBMAs, particularly when various types like analog, semi-analog, and non-analog are offered together, are appealing enough to be selected in two out of three cases when no meat option is available. Interestingly, among the three types of PBMAs, no unanimous picture of consumer preferences emerged. The semi-analog and non-analog burgers would have similar market shares if offered exclusively but also if offered together. The analog burger was less popular but not far behind the other two. The finding can help explain why some research indicates that consumers prefer alternatives that closely resemble meat in flavor, texture, appearance, and smell (35), whereas others find that consumers prefer lightly processed options that do not resemble meat over meat replacements (14). Indeed, PBMAs appear to share commonalities that allow spillover effects. For example, consideration and preference for one PBMA type increases consideration and preference for others.

Second, the availability of a meat option dramatically shifts choice, with three-quarters of individuals opting for meat over PBMAs. We find heightened PBMA preference among consumers with a college degree as well as female consumers who prefer the semi-analog burger. By contrast, consumers who self-report never eating PBMAs find extremely little utility in eating PBMAs, including those that mimic meat. Yet we observe that a majority of consumers would at least consider a PBMA option alongside meat even if they ultimately choose meat. One may speculate that a person who chooses meat after considering a PBMA, might opt for the PBMA on a subsequent occasion. Additionally, consumers who consider both meat and non-meat options may be more open to hybrid products that combine meat and plant-based ingredients. Supporting this, a study among 99 UK consumers found that after trying beef, plant-based, and hybrid burgers, acceptance for the hybrid burger was greater than that of the plant-based burger in both blind and informed conditions (36).

Third, a potentially unintended consequence of spillover effects is partial substitution among PBMAs. In study 1’s market share simulations (*SI Appendix*, Table S3), we observe that the market share for each PBMA type would increase by at least 5% points, if the other PBMAs were not available. We further observe diminishing returns of adding more PBMA variety, which could prevent these options from becoming profitable. These findings corroborate the concerns of a growing number of scholars (33–37). While many assume that stimulating consumption of PBMAs will lead to a reduction in meat consumption, this substitution pattern indicates that these products are not currently fulfilling their sustainability goal of displacing meat on a large scale. Still, as we introduce PBMAs into the choice set (study 1), there is a discernible shift in overall market share, with an increase in the PBMA share and a corresponding decrease in the meat share. Further, reducing PBMA prices “steals” meat choices but does not harm the choice of the remaining PBMAs (study 2). While our study’s methodology and design differ, this trend aligns with the observations made by Garnett and colleagues, where increasing vegetarian options in a university dining hall boosted vegetarian sales and reduced meat sales (37).

A fourth finding is that prices affect demand for PBMAs. When we factor in pricing, the preference for PBMAs falls below 20% if they are priced higher than meat, which is typically the case. If PBMAs and meat are priced equally, the preference for PBMAs increases to 21%, mirroring findings from study 1 and previous research (16, 17, 28). A further reduction in PBMA prices can significantly boost their popularity. For instance, if PBMAs are priced at half the price of meat, their choice shares increase to 38%, and nearly 50% among male consumers. However, it is important to note that these figures are based on a combined offering of various PBMA types. For restaurants and producers of PBMAs, price-elastic demand means that revenues would actually increase when they offer different PBMA types at lower prices than the meat options. Importantly, we show that price variation does not only impact choice but also consideration set size and composition. This means that even if initiatives to promote PBMA consumption may not show desired outcomes right away, the typically unnoted benefit of increased consideration could be accomplished.

Our studies have several strengths. We use two large samples representative of the United States to explore preference patterns for multiple PBMAs along the consumer consideration and choice stages. Notably, our unique contribution lies not only in the subject matter but also in the innovative methodology employed. In both studies, we were concerned with complex substitution patterns, particularly between meat and PBMA burgers. In study 1, we addressed this with a flexible specification for correlated unobserved heterogeneity. To obtain more information from each respondent, we opted for burger ranks as input for our exploded logit model. In study 2, we explicitly added burger prices as drivers for both decision stages, consideration and choice. We refrained from using the standard approach in choice-based conjoint (CBC) analysis that asks consumers to make repeated choices. Although within-subject designs allow the researcher to model unobserved preference heterogeneity, their predictive validity can drop as participants utilize decision heuristics (38, 39). As we did not want to measure price effects within respondents but in a purely between-subjects design, we opted for a simpler multivariate logit model at the consideration stage that still can account for interdependencies between alternatives without needing multiple observations per respondent. As our results show, modeling consideration and choice separately is crucial, as prices affect both decision stages resulting in complex elasticity patterns that simple models assuming full consideration at the choice stage would not be able to infer. Similar result can be found in an online shopping context (30). Finally, working with probability models and Bayesian estimation methods allows us to easily perform simulations that include the full uncertainty from the estimation.

However, our studies also have limitations that provide opportunities for future research. To gain an in-depth understanding of substitution patterns and price response, we focused on a single product category (burgers) and large samples from a single country (United States) under hypothetical conditions. While this decision benefits internal validity, we are careful to claim generalizability to other product categories and countries. This further includes the visual presentation of our stimuli, where AI was used to create images of an analog burger that convincingly looked like a meat burger. However, commercial products have struggled to achieve such visual parity with animal-based burgers. Thus, our estimates of preferences for meat analogs could be on the optimistic end of the spectrum. Future research could investigate a more diverse set of countries and products, including novel and ostensibly more sustainable animal-based proteins such as cultured meat. Ideally, these studies would be able to incorporate revealed preferences in addition to using hypothetical choice. A CBC analysis with repeated measurement that accounts for rich unobserved heterogeneity (with regard to consideration sets and preferences) would allow for flexibly estimating individual-level price parameters for targeted promotions and product recommendations. Such an application has practical relevance for online food delivery services that have detailed information about their customers and can reach them at the individual level via apps.

Future research might also explore the diverse nutritional profiles among PBMAs, which correspond to the variety of product offerings. Traditional vegetarian alternatives are generally made from whole legumes and grains, offering a nutrient-dense, low in saturated fat option with health benefits. Others, like meat analogs, can be categorized as ultraprocessed foods [though proponents argue they differ from other foods in this category, such as soda and confections (40)]. While evidence on the health value of meat analogs and their ability to replicate the nutritional profile of meat equivalents is limited, some studies suggest these foods can be associated with positive health outcomes, and processing whole-plant foods into protein isolates may not necessarily compromise their health value. Processing can improve product safety and enable fortification and enrichment (41). For instance, the Nutrition Facts Label on many of these meat-mimicking products matches real meat in levels of protein and B12, offering a comparable nutritional profile.

Another fruitful area for future research is how the marketing of PBMAs can increase preference beyond price effects. An interesting conversation in this regard is about which benefits of PBMA consumption should be highlighted to make them more competitive vis-a-vis meat. Some researchers argue that the health and sustainability advantages should be highlighted because this is the key relative strength of PBMAs (42). Other researchers, however, argue that the good taste of PBMAs should be highlighted, because many consumers expect PBMAs to be less tasty than meat (43, 44). Against this background, it has been found that advertised benefits should match an active eating goal, such as promoting sustainability when a sustainability goal is active but taste when a hedonic goal is active (45).

### Implications.

In the face of the pressing need to curtail animal consumption for environmental sustainability, our research investigates the complexities of the plant-based meat alternative (PBMA) market, particularly in the United States, one of the highest beef-consuming countries globally, with a per capita consumption of 25.32 kg in 2023 (3). Our studies uncover significant heterogeneity in the US market for PBMAs, with varying personal preferences and types showcasing diverse tastes. A substantial portion of consumers considering meat are reluctant to consider plant-based alternatives, posing a challenge for behavior change (for a similar finding, see ref. 46). However, leveraging competitive PBMA prices can enhance the likelihood of consideration, translating into greater choice. Importantly, increased consideration for one PBMA extends to other PBMAs, emphasizing the interconnected nature of these products. The association between PBMA types, combined with a strong preference for meat, also points to diminishing returns of meat displacement when the variety of available PBMA options would increase. Rather, the outcome would be comparable to microtargeting, with each PBMA attracting a specific consumer type. While this would make consumers of PBMAs happier overall, PBMA availability alone does not seem capable of attaining the goal of a sustainable protein transition. Our findings challenge the notion that PBMAs will naturally replace meat, instead aligning with recent findings that meat and meat analogs may sometimes complement each other rather than serve as substitutes (13, 32, 34, 47, 48). However, this dynamic could shift dramatically with more competitive PBMA pricing, potentially turning these products into true meat substitutes. This is particularly true for analogs, which appear to benefit the most from increased affordability.

In light of retail prices posing a challenge for PBMA adoption, and despite their lower per-gram protein production costs compared to meat, our research gives perspectives on future pathways. In part, if these higher prices are related to processing costs (7), there is optimism that as economies of scale are realized, the reduced production cost will equalize the playing field between PBMAs and their meat counterparts (49). Realization of economies of scale may take some time, as PBMAs still had a 20% price premium over beef in 2023 (18). In the long run, the lower production and processing cost of plant proteins could translate to not just equal but lower prices than meat. Nonetheless, bringing PBMAs into the consideration set and shopping baskets demands substantial price reductions. While these reductions can be revenue-increasing with elastic demand, restaurants and PBMA producers might be reluctant to reduce prices substantially. At the same time, some countries (e.g., Germany, Netherlands, and Denmark) are contemplating a meat tax, though politically difficult to implement, a policy intervention anticipated to exert significant steering effects on consumers (50). Some scholars propose allocating tax revenues toward subsidizing plant-based foods, a measure which would further address reluctances of producers (51). Such measures could heighten the relative economic appeal of PBMAs even at moderate price reductions, allowing to position PBMAs as sustainable, moderately priced alternatives to more expensive conventional meat options (34) Importantly, however, our findings highlight the importance of affordability beyond price parity in catalyzing the shift toward plant-based diets.

## Methods

The research was approved by the Martin Luther University Halle-Wittenberg ethics committee. Participants gave their informed consent at the beginning of each study.

### Participants.

Across two studies, 2,126 participants (1,196 female, 879 male, 40 nonbinary, and 11 declined to answer), aged 18 to 86 (18 to 24 = 189; 25 to 44 = 1,134; 45 to 64 = 637; Over 65 = 166), were recruited from Prolific (N_STUDY1_ = 1,003; N_STUDY2_ = 1,123). Recruitment was limited to the United States, and participation in study 1 precluded participation in the subsequent study. Across both studies, 2 participants were excluded for inconsistent response patterns, such as choosing meat while stating never to eat meat, and one participant was excluded for unauthorized repeated participation.

## Materials

Each study presented four burger alternatives: meat (beef burger), analog (plant-based burger), semi-analog (veggie burger), or non-analog (falafel burger). We decided to simply name the analog burger “plant-based burger,” which is not uncommon for meat-mimicking burgers. Veggie burgers do not try to replicate the taste and texture of meat, although the visual appearance is similar. Hence, we named the semi-analog burger accordingly. As a non-analog burger, we went with a falafel burger as it is “borrowing” its patty from another popular, traditional vegan dish. Although the falafel burger is not widely available in Western restaurants, fast-food chain Shake Shack offers a falafel burger in the Middle East.

As burger quality varies and inconsistent quality inferences may affect stated preferences across both studies (e.g., comparing a high-quality meat burger with a low-quality nonmeat burger, or vice versa), we provided images that looked appealing and stated the typical price point ($10) for the burgers to signal a medium-to-high quality level (*SI Appendix*, Figs. S1 and S3). We pretested burger images with an independent sample through Prolific (N_PRETEST_ = 100). All images were created with Microsoft Designer’s image generator to maintain a comparable appearance. Because we needed the analog burger to look like a meat replica, and the respective AI-generated images did not look like a realistic meat burger, we generated two images of a beef burger, one of which would then be used as the depiction of a meat-mimicking plant-based burger. We pretested both images to examine whether they were perceived differently. Because pretest participants evaluated both images the same, we used one image for the analog burger in the main studies and the other image for the meat burger. Below each image, we listed patty ingredients taken from commercially available products.

Pretest participants saw 3 images (semi-analog, non-analog, and one of the two analog burger images) and rated each image on how similar to meat they expected the burger to be in terms of taste, texture, and look. As expected, both versions of the analog burger were perceived as being more similar to meat (M_ANALOG_ = 5.34; M_SEMI-ANALOG_ = 2.44; M_NON-ANALOG_ = 2.36; *P*s < 0.001), while no difference was observed between the two images of the analog burger (*P* = 0.60). Participants also rated how appealing they found each option, with the majority (between 64% and 78%) stating the burgers looked (somewhat) appealing.

### Procedure.

In both studies, participants were asked to imagine they were at a hamburger restaurant for lunch and that the daily menu listed four burger options that were all the same size. In contrast to previous research (22), we did not instruct participants to imagine that “all burgers taste the same” and “have roughly equivalent nutritional content,” because it is consumers’ varying perceptions about these issues that shape their preferences. For example, there is robust evidence that consumers expect meat burgers to be tastier than nonmeat burgers (28, 52). As we examined price effects in study 2, we added a sentence to provide a price anchor (“Typically, burgers cost around $10 (fries included)”).

#### Study 1.

After the description of the setting, respondents were first asked which option they preferred for lunch. On a new page, they were asked to imagine the option was already sold out and state their second preference (we displayed the remaining three options). On yet another page, we asked respondents to indicate which of the remaining two options they preferred to get a full ranking of all options sequentially. After the ranking, we asked respondents to indicate, for each burger option, if they would genuinely consider purchasing it. After these tasks, we collected demographic information.

#### Study 2.

Participants were randomly assigned to 1 of 9 conditions as part of a 2 (relative price: 25% higher vs. lower than the baseline price) × 4 (burger option) plus baseline condition between-subjects design. In the baseline condition, all four burgers cost $10. In the remaining conditions, the price was changed to either $12.50 (higher relative price) or $7.50 (lower relative price) for one burger at a time, while the remaining three burgers had a price of $10 (*SI Appendix*, Table S4 lists burger prices at selected hamburger restaurant chains). To increase the realism of the study, we presented the burgers in ways similar to restaurant menus with a short display of key burger ingredients, rather than a full ingredient list of the patty. Because the ingredients were not provided, naming one option the “plant-based burger” may have irritated participants making them wonder whether the veggie and falafel burgers may not be plant-based. In response, we named the meat analog burger as a burger with a “tastes-like-meat” patty.

Across conditions, participants were first asked to select any and all burgers that they could imagine ordering for lunch. In a funneled presentation, participants were then asked to choose which of the previously selected options they preferred the most. Participants subsequently stated their purchase likelihood for the respective choice on a 7-point rating scale (*SI Appendix*, Fig. S3). After these tasks, we collected demographic information.

### Weighting.

To gain samples representative for the adult US population, we weighted responses based on gender identity, age, and education (*SI Appendix*, Tables S1 and S5). We note that results did not substantially vary from an analysis without weights.

### Analyses.

We provide an extended technical appendix in *SI Appendix*. All analyses were performed in Stan and R.

#### Study 1.

We estimate a hierarchical exploded logit model (24, 25) to incorporate multiple-ranked choices for each person (not just the first choice), consideration, and individual heterogeneity. Based on this model, it is possible to infer realistic counterfactuals that can be used to simulate market shares across “what if” scenarios.

#### Study 2.

In a two-stage model, we first estimate a multivariate logit model (31) to capture the interdependencies among burger alternatives, the influence of prices, and the effect of observed heterogeneity at the consideration stage and then multinomial logit model for burger choice (25), conditioned on the observed consideration sets (i.e., for each respondent, only the considered burgers enter the choice model). Combining both stages, the final (unconditional) burger choice probability is calculated by multiplying the consideration set probabilities with the conditional choice probabilities (30, 53). As we explicitly collect the consideration set and choice information, both stages are easy to model and estimate. Note that both stages do not share parameters and can be separately estimated (30) to accommodate flexible substitution patterns.

## Supplementary Material

Appendix 01 (PDF)

## Data Availability

Anonymized data and code are freely available online at the Open Science Framework (https://doi.org/10.17605/OSF.IO/EGTUQ) (54).
