# Supplementary material for: Substitution patterns and price response for plant-based meat alternatives
Source: Proc Natl Acad Sci U S A. 2024 Dec 2;121(50):e2319016121. doi: 10.1073/pnas.2319016121 (PMC11648651; doi:10.1073/pnas.2319016121)
Supplement: Supplementary file 1 — Appendix 01 (PDF) [file pnas.2319016121.sapp.pdf]

PNAS

Supporting Information for

Substitution patterns and price response for plant-based meat alternatives

Steffen Jahn, Daniel Guhl and Ainslee Erhard

Corresponding Author: Steffen Jahn
E-mail: steffen.jahn@wiwi.uni-halle.de

This PDF file includes:

Supporting text
Figs. S1 to S7
Tables S1 to S6
SI References

Supporting Information Text

Study 1. For study 1 we, use a hierarchical (or mixed) exploded logit model on the burger rankings (see (1), chapter 7.3 for details). The model is well-suited to our setting as we collected each respondent's full ranking of all J burger alternatives. It is also easy to incorporate observed and unobserved heterogeneity.

The utility of burger j for respondent i is:

$$u_{ij} = \beta_{ij} + \epsilon_{ij}, \quad \text{where} \quad \epsilon_{ij} \sim EV(0, 1). \tag{1}$$

β_{ij} is the respondent-level burger preference and ϵ_{ij} is the error term. The extreme value distribution assumption of ϵ_{ij} leads to a simple expression ("exploded logit") for the probability of a specific ranking of the J alternatives based on the utility values (see (2)):

$$Pr(u_{i1} > u_{i2} > \cdots > u_{iJ-1} > u_{iJ}) = \prod_{j=1}^{J} \frac{\exp(\beta_{ij})}{\sum_{k=j}^{J} \exp(\beta_{ij})}. \tag{2}$$

For the respondent-level burger preferences $\beta_i = [\beta_{i1}, \ldots, \beta_{iJ}]'$, we assume a "full" multivariate normal distribution (i.e., a multivariate regression specification, see (3)):

$$\beta_i = \Gamma' x_i + \eta_i, \quad \text{where} \quad \eta_i \sim MVN(0, \Sigma). \tag{3}$$

The matrix Γ includes the parameters to be estimated that relate for each respondent i observed heterogeneity (e.g., demographics) in vector x_i to the burger preferences. A special case of the model without observed heterogeneity would be $\beta_i = b + \eta_i$, where b is simply a vector with population-level average burger preferences.

In addition to the ranking, we ask consideration questions for all alternatives for an "anchoring" of the absolute value of the utilities (4–6). To this end, we have for each $j \in 1, \ldots, J$:

$$v_{ij} = \beta_{ij} + \nu_{ij}, \quad \text{where} \quad \nu_{ij} \sim \mathrm{EV}(0, 1/\mu). \tag{4}$$

The probability of the binary consideration choice (cs) for burger j of respondent i is:

$$Pr(cs_{ij} = 1) = \frac{\exp(\mu \cdot \beta_{ij})}{1 + \exp(\mu \cdot \beta_{ij})}. \tag{5}$$

Hence, we assume that the ranking utilities are the same as the consideration utilities (up to a scale factor μ). The anchor for not considering an alternative is, therefore, 0.

The full likelihood of the model is $l_i^{\text{full}} = (l_i^{\text{rank}} \times l_i^{\text{anchor}})^{w_i}$, and w_i is the normalized survey weight of respondent i. For the hierarchical prior we use a multivariate normal distribution $p(\beta_i) = MVN(b, \Sigma)$, where $\Sigma = \mathrm{diag}(\sigma) \cdot \Omega \cdot \mathrm{diag}(\sigma)$ and $p(b) = N(0, 2.5)$, $p(\sigma) = N_+(0, 1)$. The multivariate normal distribution is common for modeling heterogeneous preferences in choice modeling (1). The factorization of the covariance matrix Σ into a vector of standard deviations σ and a correlation matrix Ω is advocated in the current literature on Bayesian hierarchical models (7). The correlation matrix Ω allows the model to capture dependencies between burgers that cannot be fully explained by the observed heterogeneity in x_i. For the correlation matrix, we employ the LKJ distribution (8) as prior and shrink the correlation slightly toward zero ($p(\Omega) = LKJ(2)$). This approach has advantages over the common Wishart prior on Σ (e.g., no correlation between components of the covariance matrix; (9)). Lastly, we use a lognormal prior on μ to ensure that the scale parameter is strictly positive ($\log(\mu) \sim N(0, 0.2)$).

There is no available implementation of the model in standard statistical software. Thus, we implemented the model in the probabilistic programming language Stan (10). Furthermore, we use Hamiltonian Monte-Carlo and the No-U-Turn sampler (11) to draw samples from the posterior distribution. We call our Stan program using the `R`-package `rstan` in RStudio (12). Specifically, we run the sampler with five chains for 5,500 iterations with 500 iterations for warm-up and keep every fifth draw. This gives us 5,000 draws for each parameter for posterior inference. Chains converge quickly, are stable, and have reasonably high effective sample sizes ($n_{\text{eff}} >$ 2,500). All values for the Gelman-Rubin statistic are close to 1 (13). We also visually inspected trace plots and can confirm convergence and good mixing.

For the counterfactual simulations, we use the posterior draws of β_i and μ. Specifically, we first use the binary consideration probabilities to derive the probability for each possible consideration set. The counterfactual scenarios differ in the available set of alternatives in this step. Next, we compute the conditional choice probabilities from the logit model given each possible consideration set s (i.e., the probability for rank 1 of an alternative). The unconditional choice probabilities follow from weighting the conditional choice probabilities by the corresponding consideration set probabilities ($\mathrm{Pr}_{is} = \prod_{j \in s} Pr(cs_{ij} = 1) \cdot \prod_{j \notin s} (1 - Pr(cs_{ij} = 1))$). We aggregate over the individuals by using the survey weights to obtain choice shares. Repeating these steps for each posterior draw provides the full posterior distribution of the counterfactual simulation.

Study 2. We use a two-stage model (stage 1: consideration, stage 2: choice) for our discrete choice analysis using a between-subjects design. Two-stage models have a long history in marketing, psychology, and economics (see (14) for an overview of such models using scanner panel data and (15) for state preference data). We evaluated the pros and cons of standard choice-based conjoint (CBC) analysis, and our approach concluding the two-stage model is best suited to address our research goal of understanding the effect of prices on burger decisions while explicitly including the consideration stage. As our approach may seem like an unusual design, we will briefly describe our decision-making process:

1. A typical assumption for standard CBC is that all alternatives shown are relevant (i.e., are being considered). If consumers ignore some of the alternatives in the shown choice sets, any inference about preferences under the "full" consideration assumptions will be biased (16). Specifically, irrelevant alternatives in choice sets lead to an attenuation bias; the impact of attribute changes such as price will be underestimated (15). Based on findings of study 1, we should expect irrelevant alternatives, such as the meat burger for non-meat eaters or any PBMA burger for "meat lovers" when prices are higher than that of the meat burger.

2. Asking consumers repeatedly about their choices allows modeling unobserved preference heterogeneity and, in addition, could provide information about (unobserved) choice set heterogeneity (15, 17, 18). The potential downside is that many choice tasks are necessary to infer unobserved consideration sets from choices alone (typically more than 10 choice tasks; (15, 17)).

3. Asking many times might then lead to problematic results because respondents pick up simplification strategies and adapt their decision-making process. Li and colleagues show that a CBC study's external validity drops as early as after 3–6 choice tasks (19). This even holds for CBC studies with incentive-alignment but less so for adaptive designs (as used by Sawtooth Software) (20). On the other hand, adaptive designs that maximize statistical efficiency can be cognitively taxing to respondents (19).

4. Another option, compared to a standard CBC setup with an adaptive design and a much more complicated model that allows for unobserved choice set heterogeneity, would be to ask respondents multiple times to state their consideration set in different pricing scenarios (and then continue each time with a choice task conditional on the consideration set). We opted against this because being asked multiple times about product choice is established and common for respondents/consumers as this mimics real life. However, being asked about consideration is unusual. This process tends to involve simple decision heuristics to alleviate the cognitive burden (21), which makes it difficult for consumers to explicitly trace the dynamic consideration set formation across conditions. Consumers are typically not required to explicitly build a consideration set when making purchases (exceptions might be online configurators, etc.). Hence, it might feel strange to respondents having to build choice sets multiple times if we only changed prices for one option at a time. Further, we were concerned about the point above regarding choice task adaptation (19). If product choices alone are problematic, adding a consideration choice task to each product choice task also adds a lot of effort for respondents.

In line with our research goal of understanding the effect of price on burger decisions while explicitly including the consideration stage, we chose a practical and straightforward approach. By using a between-subjects design with only one consideration choice and one product choice per respondent, we can efficiently sample a large number of respondents with rich demographic information. We note that this approach explicitly accounts for observed choice set heterogeneity, which we think is the most relevant type of heterogeneity we deal with, reassuring us about the feasibility of our study as it mitigates concerns about unobserved preference heterogeneity.

To model the consideration and choice stages (22), we closely follow Amano and colleagues (23). In the first stage, we model the consideration of the different burger alternatives using a multivariate logit model (24–27). Here, the dependent variable for each respondent is a 4×1 vector that indicates which burger was considered. The multivariate logit model is well-suited for modeling joint discrete decisions, as it allows for 1) interdependencies across alternatives (e.g., due to overlap in features of burgers or correlated preferences), 2) the inclusion of independent variables that may affect the consideration probability for each burger (such as price or demographics), 3) a close form solution for consideration probabilities and the model likelihood. The use of the multivariate logit model, in contrast to the independent logit model, is a crucial extension of the existing literature (14). Given our moderately small number of burgers, the total number of possible consideration sets is easily manageable ($2^4 = 16$ possible consideration sets, including the empty set), we use the multinomial logit specification across all consideration sets (25):

$$Pr(Y_i = y_i|x_i) = \frac{\exp(\mu_{y_i})}{\sum_{s_i \in S} \exp(\mu_{s_i})} \quad [6]$$

with

$$\mu_{y_i} = \sum_{j=1}^{J} y_{ij} \left(\alpha_j + x_{ij}\beta_j\right) + \sum_{k>j} y_{ij} y_{ik} \psi_{jk}. \quad [7]$$

y_i is a specific combination of considered burgers from all possible consideration sets S. x_{ij} is a vector of the respondent and alternative-specific variables (i.e., demographics and price with corresponding interactions). α_j are intercepts for each burger

and β_j contain the effect of x_{ij} on the consideration. The association parameters ψ_{jk} measure whether two burgers are more ($\psi > 0$) or less ($\psi < 0$) likely to be considered together after controlling for the effects of variables in x.

The second stage is a multinomial logit model for the burger choice conditional on the particular consideration set from the first stage. Here the choice probability of respondent i for burger j is:

$$Pr(b_{ij} = 1 | x_i, s_i) = \frac{\exp(u_{ij})}{1 + \sum_{k \in s_i} \exp(u_{ik})} \qquad [8]$$

with

$$u_{ij} = \gamma_j + x_{ij}\delta_j. \qquad [9]$$

γ_j and δ_j are the intercept and slope parameters of the multinomial logit model. We also ask for purchase intention regarding the most preferred burger option (7-point scale). This allows us to define a cutoff for inferring whether a respondent would buy the burger or opt out. Similar to study 1, this anchoring enables us to estimate the utilities for all options, and we restrict the utility for the outside option (i.e., not buying any burger) to 0. We set the cutoff to "5" meaning that we label purchase intention as a purchase if respondents state they were "somewhat likely", "likely", or "very likely" to buy the burger. Values below 5 result in an observation for the outside (no purchase) option.

When we combine both stages, we get the final (unconditional) choice probability by multiplying the consideration set probabilities with the conditional choice probabilities. Hence, the unconditional choice probability for a burger j is the weighted sum of the conditional choice probability for a choice set where j is included, with the probability of each relevant choice set as weight. As we explicitly collect the consideration set and choice information, both stages are easy to model and estimate. Note that both stages do not share parameters and could be separately estimated (23). The likelihood functions for both models are simply the likelihoods of MNL models with alternative- (price) and respondent-specific (e.g., age or gender) variables (see (1) for details). As in study 1, we use normalized survey weights in both likelihood functions. Please note that our model allows for flexible substitution patterns that do not suffer from the IIA property of the multinomial logit model (independence from irrelevant alternatives (1), as we incorporate discrete consideration set heterogeneity (14)). This is crucial given the product category we investigate, and we know from study 1 that not all burgers are considered by all consumers, and substitution patterns are complex. Moshary and colleagues make a similar argument in their analysis of firearm preferences (28).

As in study 1, we use Bayesian estimation. Combining the intercepts and slope parameters in both models into the vectors $\theta_{\text{consideration}} = (\alpha_1, \ldots, \alpha_J, \beta_1, \ldots, \beta_J)'$ and $\theta_{\text{choice}} = (\gamma_1, \ldots, \gamma_J, \delta_1, \ldots, \delta_J)'$ and stacking all association parameters into the vector ψ simplifies the notation. As priors we use $p(\theta_{\text{consideration}}, \psi, \theta_{\text{choice}}) = N(0, 10)$. We also implemented both models in Stan and used for the estimation of each model the same setup as in study 1, to obtain 5,000 draws for posterior inference. As in study 1, visual inspection and formal analysis reveal that the Bayesian estimation worked well ($n_{\text{eff}} >$ 2,500 and $\hat{R}$ close to 1).

The procedure for the counterfactual simulations closely follows the description above for study 1. The difference is now that we do not vary the availability of alternatives, but the prices. We simulate consideration set probabilities and unconditional choice probability for PBMA prices between \$5 and \$12.5 (in \$0.5 steps). We use the draws of $\theta_{\text{consideration}}$, ψ, and θ_{choice} and aggregate over the individuals by using the survey weights to obtain choice shares. Repeating these steps for each posterior draw provides the full posterior distribution of the counterfactual simulation. Note that we also use simulations to obtain arc price elasticities (ε). We simulate a \$0.05 increase and decrease from the baseline price of \$10 for each alternative (including the meat burger) separately and compute the relative changes in consideration and choice probabilities using the midpoint formula.

Beef Burger

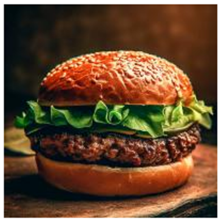

Patty ingredients:

GROUND BEEF (80% LEAN, 20% FAT), SALT, GARLIC POWDER, ONION POWDER.

Plant-based Burger

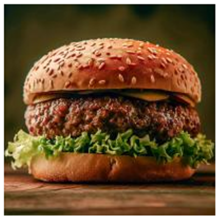

Patty ingredients:

WATER, PEA PROTEIN, CANOLA OIL, COCONUT OIL, RICE PROTEIN, FLAVORING, STABILIZER, POTATO STARCH, APPLE EXTRACT, COLOR, MALTODEXTRIN, POMEGRANATE EXTRACT, SALT, POTASSIUM SALT, CONCENTRATED LEMON JUICE, MAIZE VINEGAR, CARROT POWDER, EMULSIFIER.

Veggie Burger

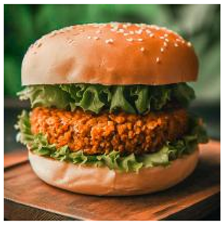

Patty ingredients:

COOKED QUINOA, RED PEPPERS, COOKED BLACK BEANS, BREAD CRUMBS, EXPELLER PRESSED CANOLA OIL, ROASTED CORN, ONIONS, POTATO FLAKES, ARROWROOT, TOMATOES, OLIVE OIL, JALAPENO PEPPERS, TOMATO PASTE, EGG WHITE POWDER, ROASTED GARLIC, WHITE VINEGAR, SALT, CILANTRO, CHILI POWDER, ONION POWDER, CORIANDER, BLACK PEPPER, GARLIC POWDER, CHIPOTLE POWDER, LIME JUICE POWDER.

Falafel Burger

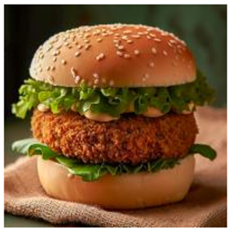

Patty ingredients:

FAVA BEANS, CHICKPEAS, SPICES, SALT, GARLIC POWDER, ONION POWDER, BAKING POWDER, DEHYDRATED PARSLEY, SUNFLOWER OIL.

Fig. S1. Stimuli (Study 1, Presentation Order was Randomized).
Note: The Beef Burger represents the meat option, while the Plant-based Burger represents the analog option. The Veggie Burger and Falafel Burger represent the semi-analog and non-analog options, respectively.

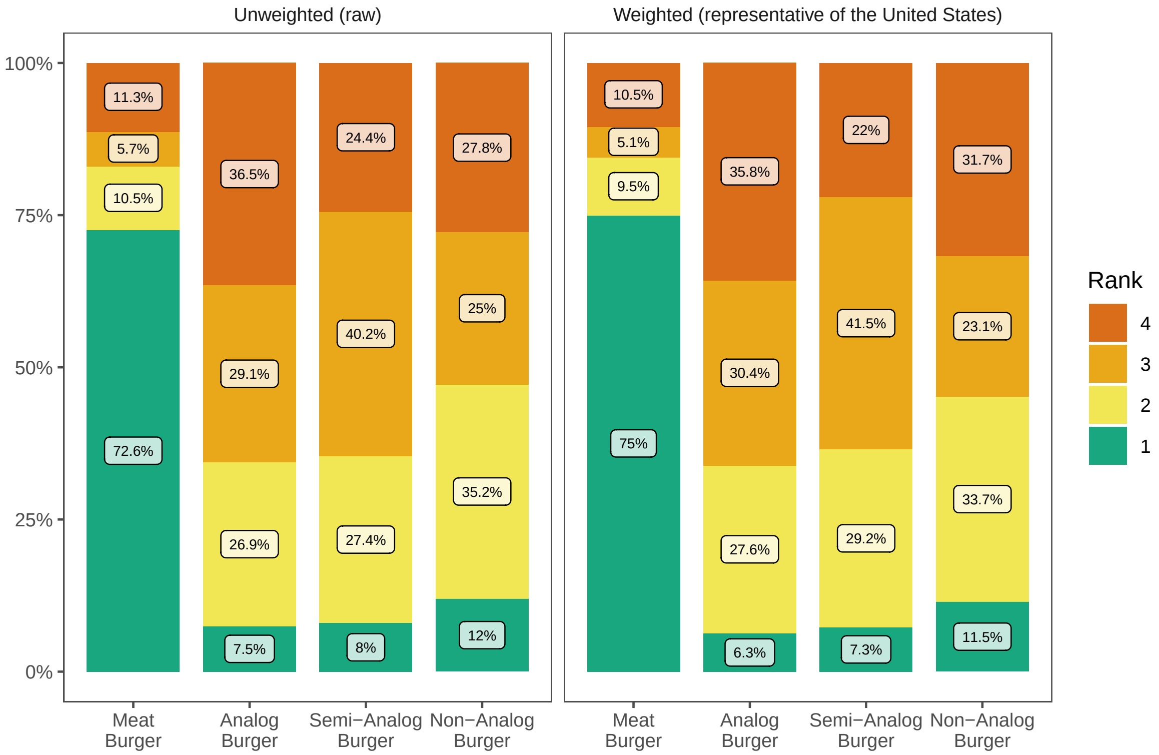

Fig. S2. Ranking of Burger Options (Study 1).

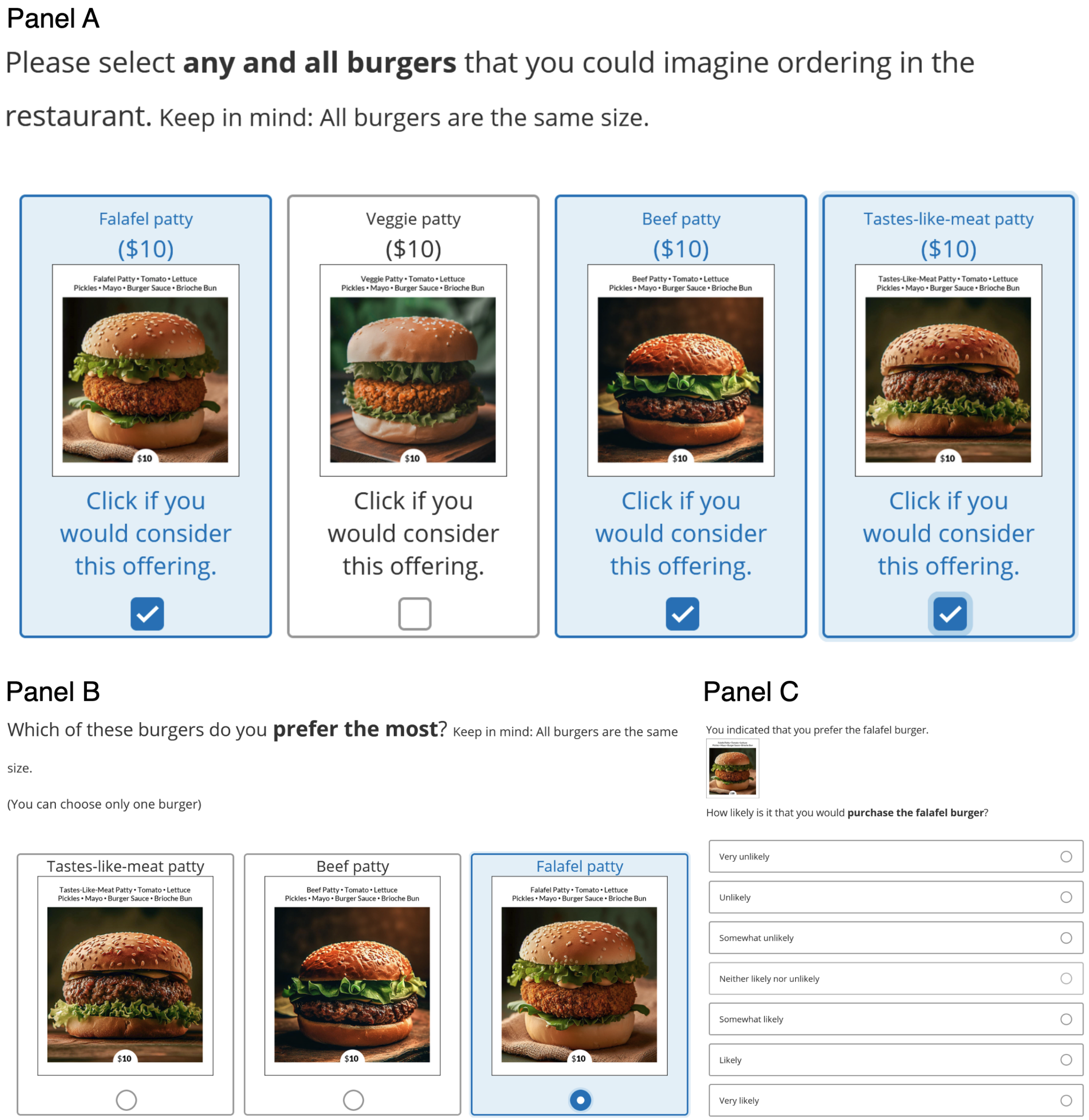

Fig. S3. Stimuli (Study 2, Baseline Condition).
Note: The figure shows the stimuli in the baseline condition (all burgers cost $10). In this example, the respondent considers three burgers (Panel *A*: meat, analog, and non-analog). Out of the three burgers, the non-analog burger is preferred the most (see Panel *B*. Panel *C* shows the follow-up question about the purchase likelihood for the most preferred burger on a 7-point scale. Please note that the burger options and corresponding images shown in Panels B and C are conditional on the specific answers of the respondent to the questions shown in Panel *A* and *B*, respectively. The order of burger options for the consideration and preference task (Panels *A* and *B*) has been randomized.

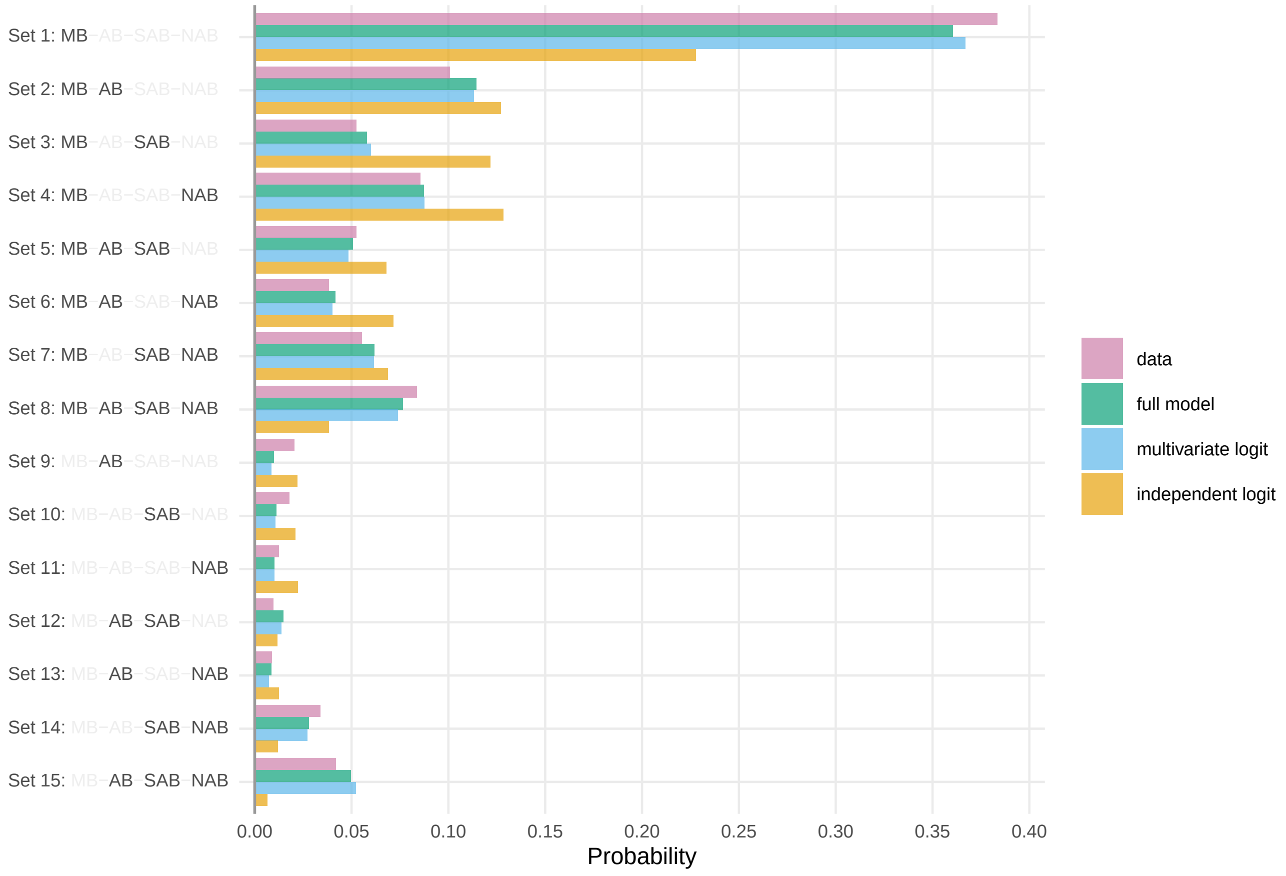

Fig. S4. Consideration Set Frequencies and Model-Based Predictions (Study 2).
Note: The figure shows the empirical frequencies and the model-based results for each possible consideration set as a unique combination of burger alternatives (MB: meat burger, AB: analog burger, SAB: semi-analog burger, NAB: non-analog burger). The independent logit with $\Pr_s = \prod_{j \in s} Pr_j \prod_{j \notin s} (1 - Pr_j)$ maps the burger probabilities (i.e., observed frequencies) to consideration set probabilities, assuming independence between alternatives. The figure shows that this model is not suited for our data as the implied consideration set probabilities and the data are significantly different ($\chi^2 = 168.1$, $df = 14$, $P < 0.01$). The data clearly shows interdependencies between burgers as, e.g., empirical frequencies for set 1 are much higher than under the independence assumption or consideration sets including the meat burger and a PBMA burger (set 2, 3, and 4) have lower empirical frequencies. Some consideration sets, including multiple PBMA burgers, have much higher frequencies than the independent logit predictions. This furnishes empirical support for complex consideration. The figure also includes the predictions of a simple multivariate logit model and the version used in the *Main Manuscript* ("full model") that includes observed heterogeneity and price effects. Both models produce results that closely align with the data (χ^2-tests are insignificant with χ^2-values < 15), showing that accounting for pairwise associations between alternatives is crucial for modeling burger consideration.

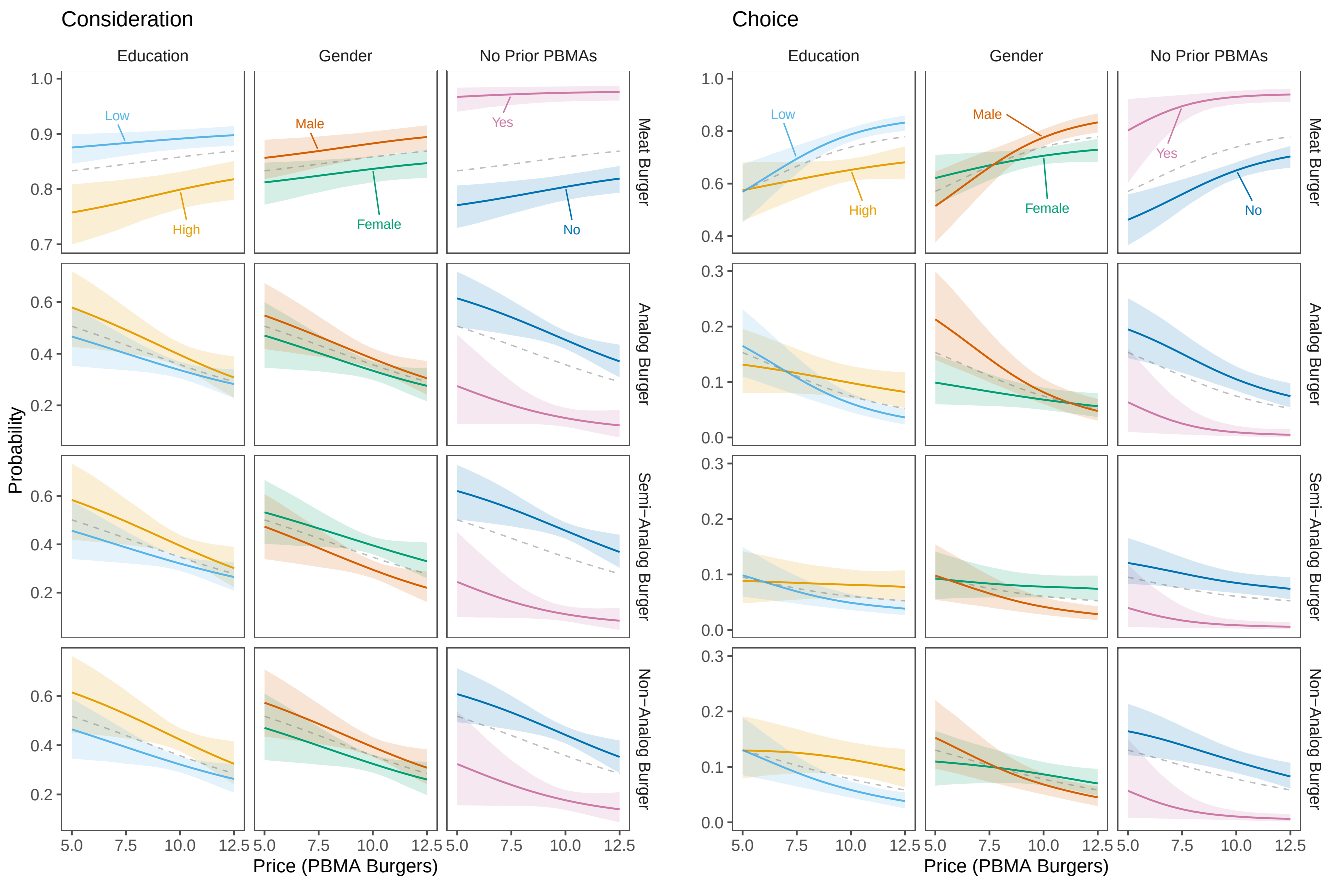

Fig. S5. Price Effect Simulations (Study 2, Part I): Education, Gender, and No Prior PBMA Eaters.
Note: Consideration and choice probabilities are shown for each burger option given a PBMA price range between $12.50 and $5, along with 95% credible intervals. Across PBMA price scenarios, the price of the meat burger was kept constant at $10. The grey dashed lines display the population mean (representative of the United States). Education (=High) refers to a college degree or higher, No Prior PBMAs (=Yes) are those who self-report to never eat plant-based meat alternatives.

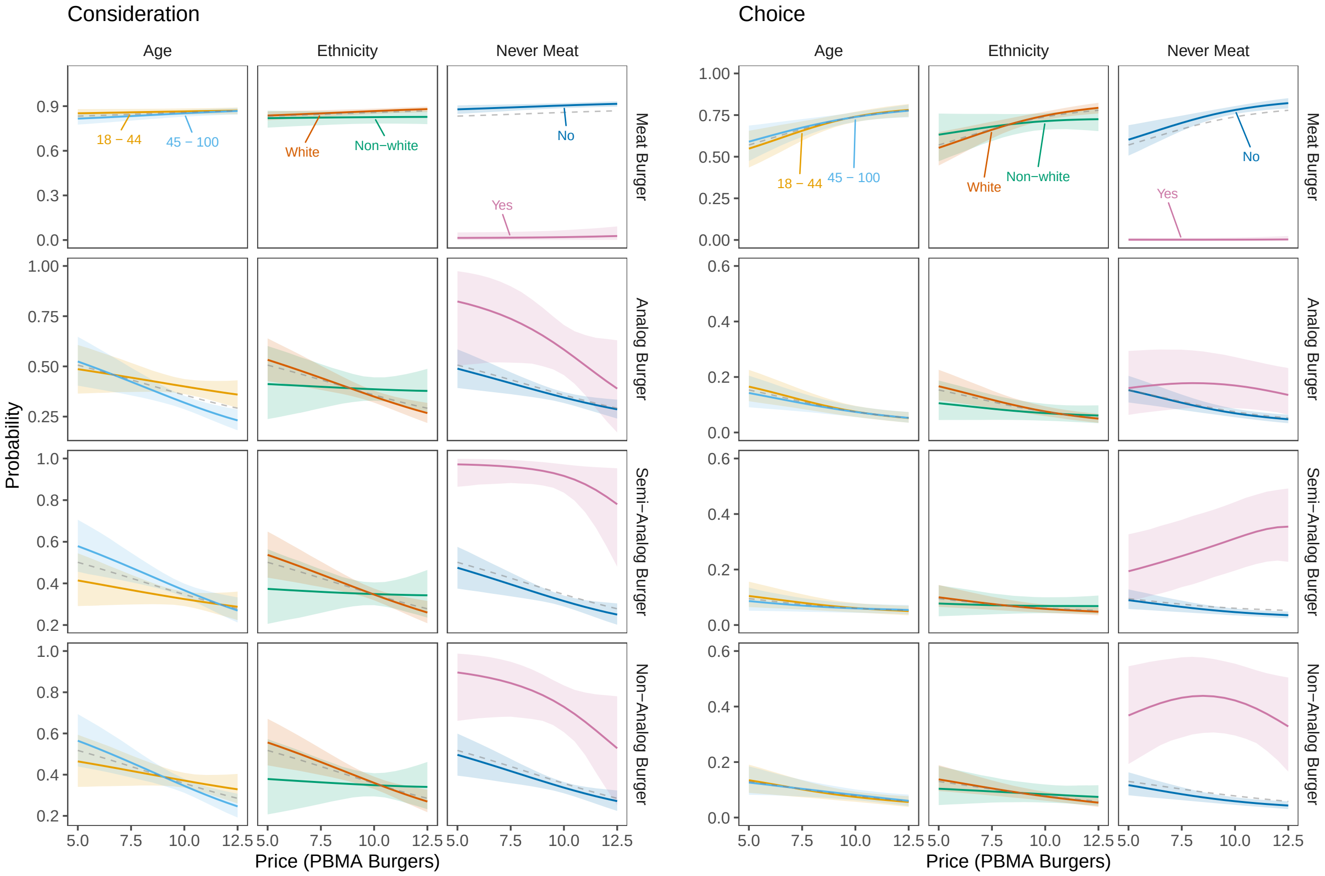

Fig. S6. Price Effect Simulations (Study 2, Part II): Age, Ethnicity, and Never Meat Eaters.
Note: Consideration and choice probabilities are shown for each burger option given a PBMA price range between $12.50 and $5, along with 95% credible intervals. Across PBMA price scenarios, the price of the meat burger was kept constant at $10. The grey dashed lines display the population mean (representative of the United States). Never Meat (=Yes) are those who self-report to never eat meat.

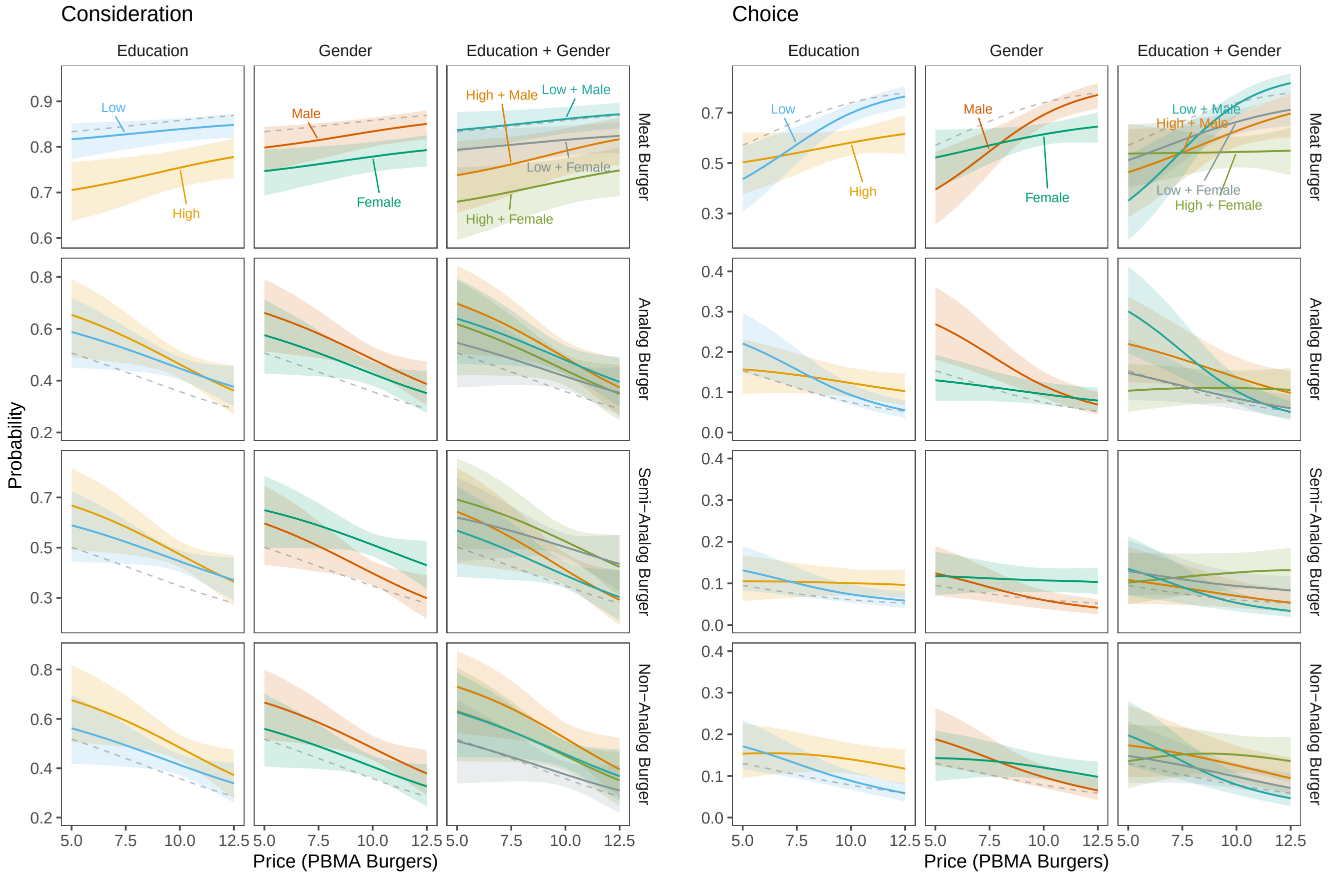

Fig. S7. Price Effect Simulations (Study 2, Part III): Education and Gender Differences After Exclusion of No Prior PBMA Eaters.
Note: Consideration and choice probabilities are shown for each burger option given a PBMA price range between $12.50 and $5, along with 95% credible intervals. Across PBMA price scenarios, the price of the meat burger was kept constant at $10. The grey dashed lines display the population mean (representative of the United States). Education (=High) refers to a college degree or higher, No Prior PBMAs (=Yes) are those who self-report to never eat plant-based meat alternatives.

Table S1. Sample Weighting (Study 1).

	Female						Male					
	Less Than Bachelor's Degree			Bachelor's Degree or Higher			Less Than Bachelor's Degree			Bachelor's Degree or Higher		
Age	Sample %	U.S. %	Weight	Sample %	U.S. %	Weight	Sample %	U.S. %	Weight	Sample %	U.S. %	Weight
18-24	2.7	4.8	1.75	2.9	0.8	0.28	1.7	5.1	2.95	1.2	0.6	0.48
25-44	13.1	9.3	0.69	15.2	7.9	0.51	12.2	10.7	0.86	13.2	7.9	0.48
45-64	9.2	10.1	1.08	7.9	6.3	0.78	4.4	10.4	2.31	5.7	6.3	0.94
Over 65	2.5	8.4	3.31	2.6	3.5	1.33	1.3	6.4	4.84	2.2	3.5	1.62

Note: Participants that could not be assigned a specific cell (N = 22) were weighted to fit the sample mean. U.S. % based on 2020 U.S. Census data (29).

Table S2. Parameter Estimates and 95% Credible Intervals (Study 1).

Panel A: Average Utility and Observed Heterogeneity

	Intercept	Age	Gender	Education	Ethnicity	Never Meat	No Prior PBMAs
Meat Burger	**4.20**	0.02	−**0.98**	−**0.70**	0.13	−**4.14**	**1.61**
	[3.67, 4.80]	[−0.14, 0.19]	[−1.50, −0.47]	[−1.21, −0.20]	[−0.46, 0.70]	[−5.22, −3.11]	[0.90, 2.36]
Analog Burger	−**1.04**	−0.10	0.07	**0.54**	0.02	**1.00**	−**3.21**
	[−1.28, −0.82]	[−0.22, 0.01]	[−0.28, 0.42]	[0.19, 0.89]	[−0.41, 0.44]	[0.07, 1.94]	[−3.82, −2.66]
Semi-Analog Burger	−**0.35**	0.08	**0.49**	0.11	−0.05	**3.90**	−**2.80**
	[−0.57, −0.17]	[−0.04, 0.19]	[0.16, 0.85]	[−0.24, 0.45]	[−0.47, 0.38]	[2.61, 5.32]	[−3.37, −2.27]
Non-Analog Burger	−**0.42**	−**0.20**	0.03	**0.49**	−0.29	0.13	−**1.99**
	[−0.63, −0.23]	[−0.33, −0.09]	[−0.32, 0.38]	[0.14, 0.85]	[−0.72, 0.15]	[−0.82, 1.05]	[−2.55, −1.50]

Note: Significant estimates (at 5%) are bolded. Age = +10y, Gender = female, Education = high (college degree or higher), Ethnicity = white, Never Meat = yes (self-report to never eat meat), No Prior PBMAs = yes (self-report to never eat plant-based meat alternatives).

Panel B: Unobserved Heterogeneity and Scale Factor

Heterogeneity	σ	95% CI
Meat Burger	1.97	[1.63, 2.35]
Analog Burger	1.77	[1.49, 2.08]
Semi-Analog Burger	1.82	[1.55, 2.11]
Non-Analog Burger	1.89	[1.59, 2.21]
**Scaling**	μ	95% CI
Scale factor	1.06	[0.88, 1.27]

Panel C: Correlations

Alternative	Meat Burger	Analog Burger	Semi-Analog Burger	Non-Analog Burger
Meat Burger	1			
Analog Burger	−**0.46** [−0.61, −0.29]	1		
Semi-Analog Burger	−**0.50** [−0.64, −0.34]	**0.75** [0.67, 0.82]	1	
Non-Analog Burger	−**0.41** [−0.58, −0.24]	**0.46** [0.35, 0.57]	**0.38** [0.26, 0.49]	1

Note: Parameter and 95% credible intervals (CI) in parentheses. Significant estimates (at 5%) are bolded.

Table S3. Market Share Simulations (Study 1).

	Meat Burger		Analog Burger		Semi-Analog Burger		Non-Analog Burger		Outside Option	
Scenario	Mean	95% CI	Mean	95% CI	Mean	95% CI	Mean	95% CI	Mean	95% CI
One Meat Option, No PBMA Options										
1	91.7	[90.7, 92.7]							8.3	[7.3, 9.3]
One Meat Option, One PBMA Option										
2	84.6	[83.5, 85.8]	12.6	[11.6, 13.6]					2.8	[2.1, 3.5]
3	82.1	[81.1, 83.3]			16.1	[15.1, 17.1]			1.8	[1.2, 2.4]
4	82.6	[81.3, 83.8]					14.8	[13.6 16.1]	2.6	[2.0, 3.3]
One Meat Option, Two PBMA Options										
5	79.4	[78.2, 80.7]	6.3	[5.4, 7.3]	13.2	[12.1, 14.3]			1.1	[0.7, 1.5]
6	78.9	[77.5, 80.1]	8.8	[7.8, 9.8]			11.2	[10.1, 12.4]	1.1	[0.8, 1.6]
7	77.0	[75.6, 78.3]			12.9	[11.8, 13.9]	9.4	[8.3, 10.6]	0.7	[0.4, 1.1]
One Meat Option, Three PBMA Options										
8	75.3	[73.8, 76.7]	4.8	[4.0, 5.6]	11.1	[10.0, 12.1]	8.4	[7.3, 9.4]	0.5	[0.3, 0.8]
No Meat Option, One PBMA Option										
9			38.0	[36.4, 39.5]					62.0	[60.5, 63.6]
10					45.2	[43.6, 46.8]			54.8	[53.2, 56.4]
11							44.9	[43.2, 46.5]	55.1	[53.5, 56.8]
No Meat Option, Two PBMA Options										
12			21.5	[19.8, 23.1]	35.1	[33.3, 37.0]			43.4	[41.6, 45.1]
13			25.1	[23.5, 26.7]			33.9	[32.3, 35.7]	41.0	[39.3, 42.6]
14					33.1	[31.4, 34.7]	30.7	[29.1, 32.4]	36.2	[34.4, 37.9]
No Meat Option, Three PBMA Options										
15			15.1	[13.6, 16.6]	27.3	[25.6, 29.0]	26.5	[24.9, 28.2]	31.0	[29.2, 32.8]

Note: Compared to a scenario with one meat and one PBMA options, adding a second PBMA option increases PBMA share and decreases meat share. For example, the average meat share across scenarios 2–4 is 83.1%, while it is 78.4% across scenarios 5–7, a decrease by 5.7%. Conversely, the average PBMA share increased from 14.5% to 20.6% (+42.0%). When 3 PBMA options are available (scenario 8), meat share further decreases by 4% (to 75.3%), while PBMA share increases by 18.0% (to 24.3%). Comparing scenarios 8 and 15, collective PBMA share increases from 24.3% to 68.9% (+183.5%).

Table S4. Burger Prices at Selected Hamburger Restaurant Chains in the United States.

Hamburger Chain	Meat Burger	Non-Meat Burger
Burger King	$5.79 (Whopper)	$6.69 (Impossible Whopper)
Carl's Jr.	$6.99 (Big Carl's)	Not sold
Fatburger	$8.99 (Original Fatburger)	$11.99 (Impossible Burger)
Five Guys	$10.69 (Hamburger)	Not sold
In-N-Out Burger	$6.19 (Double Double)	Not sold
Killer Burger	$10.75 (Classic)	$10.75 (Classic)
McDonald's	$4.69 (Big Mac)	$5.49 (McPlant[a])
Mooyah	$8.29 (MOOYAH Cheeseburger)	$12.29 (The Meatless Beast)
Red Robin	$13.99 (Keep It Simple)	$17.49 (Keep It Simple)
Shake Shack	$7.89 (ShackBurger)	$9.49 (Veggie Shack)
Smashburger	$7.79 (Classic Smash Burger)	$9.59 (Classic Smash Veggie Burger)
Whataburger	$5.39 (Whataburger)	Not sold

Prices as of July 2024. [a]The McPlant has been discontinued in the United States.

Table S5. Sample Weighting (Study 2).

	Female						Male					
	Less Than Bachelor's Degree			Bachelor's Degree or Higher			Less Than Bachelor's Degree			Bachelor's Degree or Higher		
Age	Sample %	U.S. %	Weight	Sample %	U.S. %	Weight	Sample %	U.S. %	Weight	Sample %	U.S. %	Weight
18-24	3.5	4.8	1.35	2.0	0.8	0.41	2.1	5.1	2.33	0.7	0.6	0.80
25-44	11.8	9.3	0.76	14.7	7.9	0.51	11.0	10.7	0.94	12.4	7.9	0.51
45-64	10.7	10.1	0.92	9.0	6.3	0.68	5.4	10.4	1.86	6.7	6.3	0.80
Over 65	2.5	8.4	3.29	2.3	3.5	1.48	1.0	6.4	5.00[a]	1.3	3.5	2.64

Note: Participants who could not be assigned a specific cell (N = 32) were weighted to fit the sample mean. U.S. % based on 2020 U.S. Census data (29).
[a] The estimated weight of 6.37 was capped at 5 (30).

Table S6. Parameter Estimates and 95% Credible Intervals (Study 2).

Panel A: Consideration

	Intercept	Age	Gender	Education	Ethnicity	Never Meat	No Prior PBMAs
Meat Burger	**3.07**	−**0.14**	−0.29	−**0.72**	**0.54**	−**6.39**	**1.54**
	[2.64, 3.52]	[−0.28, −0.00]	[−0.75, 0.17]	[−1.16, −0.28]	[0.00, 1.05]	[−9.16, −4.56]	[0.75, 2.44]
Analog Burger	−**0.71**	−**0.12**	−**0.34**	0.04	0.02	−0.15	−**1.13**
	[−1.13, −0.28]	[−0.21, −0.04]	[−0.62, −0.08]	[−0.24, 0.32]	[−0.31, 0.35]	[−0.83, 0.54]	[−1.47, −0.79]
Semi-Analog Burger	−**1.23**	**0.18**	**0.69**	−0.09	−0.05	**2.22**	−**1.41**
	[−1.72, −0.75]	[0.08, 0.28]	[0.38, 0.99]	[−0.40, 0.22]	[−0.42, 0.33]	[1.20, 3.42]	[−1.81, −1.02]
Non-Analog Burger	−**0.58**	−0.06	−**0.60**	0.25	0.21	0.13	−**0.63**
	[−1.03, −0.12]	[−0.15, 0.03]	[−0.90, −0.31]	[−0.04, 0.54]	[−0.14, 0.54]	[−0.60, 0.88]	[−0.99, −0.28]
Price (Meat)	0.17	0.02	−0.09	−0.12	−**0.58**	−0.16	0.09
	[−0.08, 0.42]	[−0.08, 0.14]	[−0.47, 0.30]	[−0.49, 0.23]	[−1.01, −0.19]	[−1.57, 1.34]	[−0.59, 0.72]
Price (PBMAs)	−**0.11**	−0.03	0.03	−0.03	−0.09	−0.19	0.01
	[−0.18, −0.04]	[−0.07, 0.01]	[−0.10, 0.16]	[−0.17, 0.11]	[−0.26, 0.07]	[−0.55, 0.14]	[−0.16, 0.18]

Panel B: Choice

	Intercept	Age	Gender	Education	Ethnicity	Never Meat	No Prior PBMAs
Meat Burger	**3.20**	0.05	−**0.86**	−0.11	0.26	−**8.82**	0.07
	[2.38, 3.82]	[−0.16, 0.27]	[−1.59, −0.15]	[−0.83, 0.61]	[−0.52, 1.00]	[−23.12, −0.37]	[−0.67, 0.83]
Analog Burger	**1.64**	0.14	−**0.85**	0.23	0.63	−0.10	−**1.65**
	[1.06, 2.20]	[−0.10, 0.38]	[−1.71, −0.03]	[−0.60, 1.06]	[−0.29, 1.58]	[−1.35, 1.29]	[−3.23, −0.30]
Semi-Analog Burger	**1.30**	−0.01	−0.36	0.36	0.30	0.34	−1.221
	[0.71, 1.90]	[−0.27, 0.23]	[−1.27, 0.50]	[−0.47, 1.23]	[−0.63, 1.20]	[−0.81, 1.65]	[−2.67, 0.19]
Non-Analog Burger	**1.45**	0.13	−0.30	0.40	0.23	0.98	−**1.64**
	[0.93, 1.97]	[−0.12, 0.38]	[−1.17, 0.56]	[−0.43, 1.23]	[−0.69, 1.14]	[−0.21, 2.32]	[−2.95, −0.42]
Price	−**0.19**	**0.08**	**0.29**	**0.35**	−0.12	**0.47**	−0.13
	[−0.34, −0.04]	[0.01, 0.15]	[0.05, 0.52]	[0.13, 0.58]	[−0.41, 0.15]	[0.08, 0.88]	[−0.52, 0.26]

Note: Significant parameter estimates (at 5%) are bolded. Association parameters are shown in the *Main Manuscript*, Table 1. Age = +10y, Gender = female, Education = high (college degree or higher), Ethnicity = white, Never Meat = yes (self-report to never eat meat), No Prior PBMAs = yes (self-report to never eat plant-based meat alternatives).

Survey Questions (Study 1)

Setting description

Imagine you go to a burger place that offers **four lunch specials**.
All burgers cost the same and are the same size.

The next page will display the four lunch specials.

Please take your time to consider all four burger options.
We will subsequently ask questions about what you think of these burgers.

Q1 Now we'd like you to rate these burgers.

For this research, it is essential that you **answer honestly**. There are no right or wrong answers and no trick questions.

Remember: **All burgers cost the same and are the same size.**

Questions 2-6 are product evaluations that were asked for each burger option. The order of burgers and questions was randomized.

Q2 How **tasty** do you consider the (beef/plant-based/veggie/falafel) burger to be?

○ (-3) very not tasty
○ (-2) not tasty
○ (-1) somewhat not tasty
○ (0) neutral
○ (1) somewhat tasty
○ (2) tasty
○ (3) very tasty

Q3 How **healthy** do you consider the (beef/plant-based/veggie/falafel) burger to be?

○ (-3) very unhealthy
○ (-2) unhealthy
○ (-1) somewhat unhealthy
○ (0) neutral
○ (1) somewhat healthy
○ (2) healthy
○ (3) very healthy

Q4 How **sustainable** do you consider the (beef/plant-based/veggie/falafel) burger to be?

○ (-3) very unsustainable
○ (-2) unsustainable
○ (-1) somewhat unsustainable
○ (0) neutral
○ (1) somewhat sustainable
○ (2) sustainable
○ (3) very sustainable

Q5 How **processed** do you consider the (beef/plant-based/veggie/falafel) burger to be?

○ (-3) very unprocessed
○ (-2) unprocessed
○ (-1) somewhat unprocessed
○ (0) neutral

○ (1) somewhat processed
○ (2) processed
○ (3) very processed

Q6 How **authentic** do you consider the (beef/plant-based/veggie/falafel) burger to be?

○ (-3) very inauthentic
○ (-2) inauthentic
○ (-1) somewhat inauthentic
○ (0) neutral
○ (1) somewhat authentic
○ (2) authentic
○ (3) very authentic

Preference

Q7 Which of these burgers do you **prefer the most**?
(You can choose only one burger)

Q8 **If the [selected] burger were sold out already**, which of the remaining would you prefer?
(You can choose only one burger)

Attention check
The color test you are about to take part in is very simple. When asked for your favorite color, you must select "yellow." This is an attention check. Based on the text above, which color have you been asked to enter as your favorite color?

○ Green

○ Yellow

○ Orange

○ Red

○ Blue

Preference (cont'd)

Q9 You have indicated your top-2 picks. Which of the remaining two would you rather prefer?

Consideration

Q10 Out of these burgers you've just seen, which ones would you **genuinely consider purchasing** (i.e., you would consider buying outside this study) and which would you not? (Burgers are presented in alphabetical order.)
Are these burgers genuine purchase options for you?

Beef burger	○ Yes	○ No
Falafel burger	○ Yes	○ No
Plant-based burger	○ Yes	○ No
Veggie burger	○ Yes	○ No

Intuitions (Question order was randomized)

Thank you for your participation so far!

In general, people have different beliefs about food. We are interested in what you personally believe is true about certain foods. Please indicate how much you agree or disagree with the following statements.

Q11 Food that is **healthy** is generally **less tasty**.

○ (-3) strongly disagree
○ (-2) disagree

○ (-1) somewhat disagree
○ (0) neither agree nor disagree
○ (1) somewhat agree
○ (2) agree
○ (3) strongly agree

Q12 Food that **tastes better** is generally **less sustainable**.

○ (-3) strongly disagree
○ (-2) disagree
○ (-1) somewhat disagree
○ (0) neither agree nor disagree
○ (1) somewhat agree
○ (2) agree
○ (3) strongly agree

Q13 Food that is **unhealthy** generally **tastes better**.

○ (-3) strongly disagree
○ (-2) disagree
○ (-1) somewhat disagree
○ (0) neither agree nor disagree
○ (1) somewhat agree
○ (2) agree
○ (3) strongly agree

Q14 Food that is **processed** is generally **less sustainable**.

○ (-3) strongly disagree
○ (-2) disagree
○ (-1) somewhat disagree
○ (0) neither agree nor disagree
○ (1) somewhat agree
○ (2) agree
○ (3) strongly agree

Q15 Food that is **processed** is generally **less healthy**.

○ (-3) strongly disagree
○ (-2) disagree
○ (-1) somewhat disagree
○ (0) neither agree nor disagree
○ (1) somewhat agree
○ (2) agree
○ (3) strongly agree

Q16 Food that is **unprocessed** generally **tastes better**.

○ (-3) strongly disagree
○ (-2) disagree
○ (-1) somewhat disagree
○ (0) neither agree nor disagree
○ (1) somewhat agree

○ (2) agree
○ (3) strongly agree

Q17 Food that is **sustainable** is generally **healthier**.

○ (-3) strongly disagree
○ (-2) disagree
○ (-1) somewhat disagree
○ (0) neither agree nor disagree
○ (1) somewhat agree
○ (2) agree
○ (3) strongly agree

Demographics

Before we conclude, please answer a few last questions about your personal information to help us better understand our respondents.

Q18 How would you describe your gender?

○ Male
○ Female
○ Non-binary / third gender
○ Prefer not to say

Q19 How old are you?

Q20 What is your ethnicity (simplified)?

○ Asian
○ Black
○ Mixed
○ Other
○ White

Q21 What is your US Zip Code?

Q22 What is the highest level of education you have completed?

○ Some high school or less
○ High school diploma or GED
○ Some college but no degree
○ Associates or technical degree
○ Bachelor's degree
○ Graduate or professional degree (MA, MS, MBA, PhD, JD, MD, DDS, etc.)
○ Prefer not to say

Q23 Which of the following best describes your diet?

○ Omnivore (I regularly eat meat/dairy products)
○ Flexitarian (I occasionally eat meat/dairy products)
○ Pescetarian (I never eat meat but I eat fish)
○ Vegetarian (I never eat meat but I eat dairy products)
○ Vegan (I never eat animal-derived products)
○ Halal/Kosher (I eat meat prepared according to Muslim/Jewish regulations)

Q24 Are you currently following a diet or actively engaged in weight management?

○ Yes
○ No
○ Prefer not to answer

Q25 How frequently do you eat meat?

○ Never
○ Rarely
○ One to three times a month
○ One to four times a week
○ Everyday or almost everyday
○ Multiple times a day

Q26 How frequently do you eat plant-based meat alternatives?

○ Never
○ Rarely
○ One to three times a month
○ One to four times a week
○ Everyday or almost everyday
○ Multiple times a day

Thank You

SI References

1. K Train, *Discrete Choice Methods with Simulation.* (Cambridge University Press), 2nd edition, (2009).
2. R Chapman, R Staelin, Exploiting rank ordered choice set data within the stochastic utility model. *J. Mark. Res.* **19**, 288–301 (1982).
3. P Rossi, G Allenby, S Misra, *Bayesian Statistics and Marketing.* (Wiley), 2nd edition, (2024).
4. T Dyachenko, R Reczek, G Allenby, Models of sequential evaluation in best-worst choice tasks. *Mark. Sci.* **33**, 828–848 (2014).
5. K Chrzan, B Orme, *Applied MaxDiff: A Practitioner's Guide to Best-Worst Scaling.* (Sawtooth Software), (2019).
6. JB Schramm, M Lichters, Incentive alignment in anchored MaxDiff yields superior predictive validity. *Mark. Lett.* (2024) Advance online publication: https://link.springer.com/article/10.1007/s11002-023-09714-2 [Accessed August 4, 2024].
7. R McElreath, *Statistical Rethinking: A Bayesian Course with Examples in R and Stan.* (CRC Press), 2nd edition, (2020).
8. R Lewandowski, D Kurowicka, H Joe, Generating random correlation matrices based on vines and extended onion method. *J. Multivar. Analysis* **100**, 1989–2001 (2009).
9. D Akinc, M Vandebroek, Bayesian estimation of mixed logit models: Selecting an appropriate prior for the covariance matrix. *J. Choice Model.* **29**, 133–151 (2018).
10. B Carpenter, et al., Stan: A probabilistic programming language. *J. Stat. Softw.* **76**, 1–32 (2017).
11. M Hoffman, A Gelman, The No-U-Turn sampler: Adaptively setting path lengths in Hamiltonian Monte Carlo. *J. Mach. Learn. Res.* **15**, 1593–1623 (2014).
12. Stan Development Team, RStan: the R interface to Stan (2023) R package version 2.32.5.
13. A Gelman, et al., *Bayesian Data Analysis.* (CRC Press), 3rd edition, (2013).
14. A Manrai, R Andrews, Two-stage discrete choice models for scanner panel data: An assessment of process and assumptions. *Eur. J. Oper. Res.* **111**, 193–215 (1998).
15. J Louviere, D Hensher, J Swait, *Stated Choice Methods.* (Cambridge University Press), (2000).
16. J Swait, *Probabilistic Choice Set Generation in Transportation Demand Models.* (Ph.D. Thesis, Massachusetts Institute of Technology), (1984).
17. J Abaluck, A Adams-Prassl, What do consumers consider before they choose? identification from asymmetric demand responses. *Q. J. Econ.* **136**, 1611–1663 (2021).
18. J Chiang, S Chib, C Narasimhan, Markov chain Monte Carlo and models of consideration set and parameter heterogeneity. *J. Econom.* **89**, 223–248 (1998).
19. Y Li, et al., The more you ask, the less you get: When additional questions hurt external validity. *J. Mark. Res.* **59**, 963–982 (2022).
20. V Sablotny-Wackershauser, M Lichters, D Guhl, P Bengart, B Vogt, Crossing incentive alignment and adaptive designs in choice-based conjoint: A fruitful endeavor. *J. Acad. Mark. Sci.* **52**, 610–633 (2024).
21. A Aouad, V Farias, R Levi, Assortment optimization under consider-then-choose choice models. *Manag. Sci.* **67**, 3368–3386 (2021).
22. C Manski, The structure of random utility models. *Theory Decis.* **8**, 229–254 (1977).
23. T Amano, A Rhodes, S Seiler, Flexible demand estimation with search data (2022) Available at SSRN: https:/doi.org/10.2139/ssrn.3214812 [Accessed August 4, 2024].
24. J Besag, Spatial interaction and the statistical analysis of lattice systems. *J. Royal Stat. Soc. Ser. B (Methodological)* **36**, 192–225 (1974).
25. K Bel, D Fok, R Paap, Parameter estimation in multivariate logit models with many binary choices. *Econom. Rev.* **37**, 534–550 (2018).
26. G Russell, A Petersen, Analysis of cross category dependence in market basket selection. *J. Retail.* **76**, 367–392 (2000).
27. V Caputo, JL Lusk, D Blaustein-Rejto, Plant-based versus conventional meat: Substitution, complementarity, and market impacts (2023) Available at: https://static1.squarespace.com/static/502c267524aca01df475f9ec/t/6526c7450f0d7e4a89d8c158/1697040198414/Manuscript+-+PBMAs+-FINAL.pdf [Accessed August 13, 2024].
28. S Moshary, B Shapiro, S Drango, Preferences for firearms and their implications for regulation (2023) Available at SSRN: https://ssrn.com/abstract=4194121 [Accessed October 7, 2024].
29. United States Census Bureau, Census data (2020) Available at: https://data.census.gov/ [Accessed November 28, 2023].
30. JP Helveston, et al., Will subsidies drive electric vehicle adoption? Measuring consumer preferences in the u.s. and china. *Transp. Res. Part A: Policy Pract.* **73**, 96–112 (2015).
